# Comparing polysaccharide decomposition between the type strains *Gramella echinicola* KMM 6050^T^ (DSM 19838^T^) and *Gramella portivictoriae* UST040801-001^T^ (DSM 23547^T^), and emended description of *Gramella echinicola* Nedashkovskaya et al. 2005 emend. Shahina et al. 2014 and *Gramella portivictoriae* Lau et al. 2005

**DOI:** 10.1186/s40793-016-0163-9

**Published:** 2016-06-03

**Authors:** Irina Panschin, Sixing Huang, Jan P. Meier-Kolthoff, Brian J. Tindall, Manfred Rohde, Susanne Verbarg, Alla Lapidus, James Han, Stephan Trong, Matthew Haynes, T. B. K. Reddy, Marcel Huntemann, Amrita Pati, Natalia N. Ivanova, Konstantinos Mavromatis, Victor Markowitz, Tanja Woyke, Markus Göker, Hans-Peter Klenk, Nikos C. Kyrpides, Richard L. Hahnke

**Affiliations:** Leibniz Institute DSMZ – German Collection of Microorganisms and Cell Cultures, Braunschweig, Germany; Helmholtz Centre for Infection Research, Braunschweig, Germany; Centre for Algorithmic Biotechnology, St. Petersburg State University, St. Petersburg, Russia; Department of Energy Joint Genome Institute, Genome Biology Program, Walnut Creek, CA USA; Biological Data Management and Technology Center, Lawrence Berkeley National Laboratory, Berkeley, CA USA; School of Biology, Newcastle University, Newcastle upon Tyne, UK; School of Biology, King Abdulaziz University, Jeddah, Saudi Arabia

**Keywords:** Carbohydrate active enzyme, Polysaccharide, Bioethanol, Gliding motility, Cellulose, Marine, Flavobacteriaceae, Bacteroidetes, GEBA, KMG I

## Abstract

Strains of the genus *Gramella* (family *Flavobacteriacae*, phylum *Bacteroidetes*) were isolated from marine habitats such as tidal flat sediments, coastal surface seawater and sea urchins. *Flavobacteriaceae* have been shown to be involved in the decomposition of plant and algal polysaccharides. However, the potential to decompose polysaccharides may differ tremendously even between species of the same genus. *Gramella echinicola* KMM 6050^T^ (DSM 19838^T^) and *Gramella portivictoriae* UST040801-001^T^ (DSM 23547^T^) have genomes of similar lengths, similar numbers of protein coding genes and RNA genes. Both genomes encode for a greater number of peptidases compared to ’*G. forsetii*’. In contrast to the genome of ’*G. forsetii*’, both genomes comprised a smaller set of CAZymes. Seven polysaccharide utilization loci were identified in the genomes of DSM 19838^T^ and DSM 23547^T^. Both *Gramella* strains hydrolyzed starch, galactomannan, arabinoxylan and hydroxyethyl-cellulose, but not pectin, chitosan and cellulose (Avicel). Galactan and xylan were hydrolyzed by strain DSM 19838^T^, whereas strain DSM 23547^T^ hydrolyzed pachyman and carboxy-methyl cellulose. Conclusively, both *Gramella* type strains exhibit characteristic physiological, morphological and genomic differences that might be linked to their habitat. Furthermore, the identified enzymes mediating polysaccharide decomposition, are of biotechnological interest.

**Electronic supplementary material:**

The online version of this article (doi:10.1186/s40793-016-0163-9) contains supplementary material, which is available to authorized users.

## Introduction

Strain UST040801-001^T^ (=DSM 23547^T^ = JCM 13192^T^ = NBRC 101534^T^ = NRRLB-41137^T^) is the type strain of *G. portivictoriae* [1] and strain KMM 6050^T^ (=DSM 19838^T^ =JCM 13510^T^ =KCTC 12278^T^ =LMG 22585^T^ =NBRC 100593^T^) is the types train of *G. echinicola* [2], the type species of *Gramella* [2] of the family *Flavobacteriaceae* [3, 4]. *G. echinicola* KMM 6050^T^ was isolated from the sea urchin *Strongylocentrotus intermedius* of the Sea of Japan [2], whereas *G. portivictoriae* UST040801-001^T^ was isolated from sediment of the Victoria Harbor, Hong Kong [1]. All other *Gramella* known strains were isolated from marine habitats, such as tidal flat sediment [5–8] and coastal surface seawater [9, 10]. Many *Flavobacteriaceae* have been shown to harbour a great set of carbohydrate active enzymes, such as *Zobellia**galactinovorans* [11], *Formosa agariphila* [12], ’*Gramella forsetii*’ KT0803 [13]. However, the set of CAZymes within a genus may differ tremendously, as shown for *Polaribacter* [14] and *Flavobacterium* [15, 16]. Thus, we selected these *Flavobacteriaceae* type strains from different marine habitats to gain insights into their unknown polysaccharide decomposition potential (other than starch, cellulose and chitin).

Here we present the different sets of carbohydrate active enzymes, polysaccharide-utilization loci and peptidases of both *Gramella* genomes and a summary of their current classification, the set of known phenotypic features and a description of the permanent draft genome sequence and annotation derived from cultures of strains DSM 19838^T^ and DSM 23547^T^. Furthermore, we investigated the polar lipid profiles, cell surface structures and gliding motility of these strains, as well as the hydrolysis of certain polysaccharides.

## Organism information

### Classification and features

The draft genome of *G. echinicola*DSM 19838^T^ has one full-length and one partial 16S rRNA gene sequence identical with the sequence from the original species description (AB681204, AY608409). The draft genome of *G. portivictoriae*DSM 23547^T^ has one full-length 16S rRNA gene sequence identical with the sequence from strain NBRC 101534^T^ (AB681471) and 99 % similar with the sequence in the original species description (DQ002871) [1]. Based on 16S rRNA gene sequence similarity, closely related strains were TW-JL-80 (DQ073100, 98.1 %) from the South China Sea [17], MAR_2010_163 (JX854363, 97.3 %) from the North Sea [18] and the clone Vis_St18_35 (FN433421, 98.3 %) from the North Atlantic subtropical gyre [19]. A summary of the classification and general features of *G. echinicola*DSM 19838^T^ and *G. portivictoriae*DSM 23547^T^ is shown in Table 1.

**Table 1:** Classification and general features of *G. echinicola* DSM 19838^T^ and *G. portivictoriae* DSM 23547^T^ in accordance with the MIGS recommendations [60], as developed by [25], List of Prokaryotic names with Standing in Nomenclature [61, 62] and the Names for Life database [63]

MIGS ID	Property	DSM 19838^T^		DSM 23547^T^	
		Term	Evidence code^a^	Term	Evidence code^a^
	Current	Domain *Bacteria*	TAS [64]	Domain *Bacteria*	TAS [64]
	classification	Phylum *Bacteroidetes*	TAS [65, 66]	Phylum *Bacteroidetes*	TAS [65, 66]
		Class *Flavobacteriia*	TAS [67, 68]	Class *Flavobacteriia*	TAS [67, 68]
		Order *Flavobacteriales*	TAS [4, 69]	Order *Flavobacteriales*	TAS [4, 69]
		Family *Flavobacteriaceae*	TAS [3, 4]	Family *Flavobacteriaceae*	TAS [3, 4]
		Genus *Gramella*	TAS [2]	Genus *Gramella*	TAS [2]
		Species *Gramella echinicola*	TAS [2]	Species *Gramella portivictoriae*	TAS [1]
		Type strain KMM 6050^T^	TAS [2]	Type strain UST040801-001^T^	TAS [1]
	Gram-stain	Negative	TAS [2]	Negative	TAS [1]
	Cell shape	Rod-shaped	TAS [2]	Rod-shaped	TAS [1]
	Motility	Motile, gliding	TAS [2]	Motile, gliding	TAS [1]
	Sporulation	Non-spore forming	TAS [2]	Non-spore forming	TAS [1]
	Temperature range	Mesophilic, 4–37 °C	TAS [2]	Mesophilic, 4–36 °C	TAS [1]
	Optimum temperature	23–25 °C	TAS [2]	28–30 °C	TAS [1]
	pH range; optimum	4–11, 7–8	TAS [2]	6–10, 7–8	TAS [1]
MIGS-22	Oxygen requirement	Strictly aerobic	TAS [2]	Strictly aerobic	TAS [1]
	Carbon source	Carbohydrates, peptides	TAS [2]	Carbohydrates, peptides	TAS [1]
	Energy source	Chemoheterotroph	TAS [2]	Chemoheterotroph	TAS [1]
MIGS-6	Habitat	Marine, host, sea urchin	TAS [2]	Marine, sediment	TAS [1]
MIGS-6.3	Salinity (% NaCl, w/v)	1–15 %	TAS [2]	1–6 %	TAS [1]
MIGS-15	Biotic relationship	Commensal	TAS [2]	Free-living	TAS [1]
MIGS-14	Pathogenicity	Not reported	NAS	Not reported	NAS
	Biosafety level	1	TAS [70]	1	TAS [70]
MIGS-4	Geographic location	Troitsa Bay, Gulf of Peter the Great, Sea of Japan	TAS [2]	Victoria Harbour, Hong Kong	TAS [1]
MIGS-5	Sample collection time	1. Sep. 2002	NAS	Before 2005	NAS
MIGS-4.1	Latitude	42.64	NAS	22.31	NAS
MIGS-4.2	Longitude	131.10	NAS	114.12	NAS
	Depth	3 m	TAS [2]	not reported	

^a^Evidence codes - *TAS* traceable author statement (i.e., a direct report exists in the literature), *NAS* non-traceable author statement (i.e., not directly observed for the living, isolated sample, but based on a generally accepted property for the species, or anecdotal evidence). Evidence codes are from the Gene Ontology project [71]

**Fig. 1:**
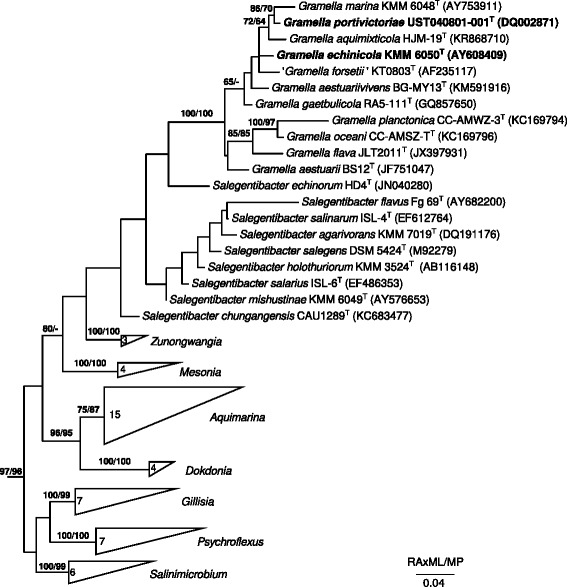
Phylogenetic tree the genus *Gramella* and closely related genera of the family *Flavobacteriaceae*. The tree was inferred from 1,409 aligned characters of the 16S rRNA gene sequence under the maximum likelihood (ML) and maximum parsimony [MP] criterion as previously described by Göker et al. [51]. The sequences of the LTP v. 121 database [52, 53] and from GenBank were aligned in ARB [54] using the SINA aligner [39] and manually corrected. The branches are scaled in terms of expected number of substitutions per site. Numbers adjacent to the branches are support values from 1,000 ML bootstrap replicates (left) and from 1,000 maximum-parsimony bootstrap replicates (right) if larger than 60 % [51]. Numbers in wedges represent the numbers of sequences. The tree was rooted using type strains of the genera *Doktonia*, *Aquimarina*, *Salinimicrobium*, *Psychroflexus*, *Gillisia* and *Mesonia*

**Fig. 2:**
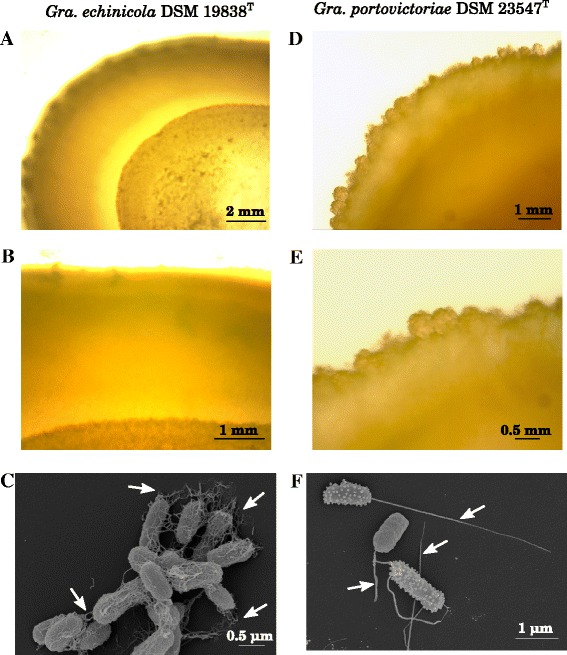
Gliding motility and scanning electron micrographs of *G. echinicola* DSM 19838^T^ and *G. portivictoriae* DSM 23547^T^. (A-F) DSM 19838^T^ and DSM 23547^T^ were incubated on bacto marine soft agar (0.3 % agar) at 25 °C to visualize the gliding motility of these *Gramella*. (G-H) DSM 19838^T^ and DSM 23547^T^ were cultured in bacto marine broth at 25 °C and visualized by scanning electron microscopy. DSM 19838^T^ expressed extracellular polymeric substances, EPS (arrows) whereas DSM 23547^T^ produced appendages (arrows)

Figure 1 depicts a 16S rRNA gene sequence phylogenomic tree of the genera *Gramella*, *Zunongwangia* and other closely related *Flavobacteriaceae*. *Gramella* spp. Nedashkovskaya et al. 2005 are Gram-stain negative, rod-shaped, strictly aerobic *Flavobacteriaceae* that are cytochrom-oxidase and catalase positive, move by gliding, produce non-diffusible carotenoid pigments, but not flexirubin-like pigments [2]. *G. echinicola*DSM 19838^T^ produces extracellular polymeric substances, whereas *G. portivictoriae*DSM 23547^T^ produces appendages (Fig. 2). Colonies of both of these *Gramella* species are circular, convex with entire translucent margins and yellow–orange in color on marine agar (Fig. 2). Both strains grow at pH 6–10 and between 4 °C and 36 °C, with a temperature optimum at 23–25 °C for *G. echinicola* and 28–30 °C for *G. portivictoriae* [1, 2]. *G. echinicola* is able to grow in medium of higher salinity (1–15 % (w/v) NaCl) than *G. portivictoriae* (1–6 % (w/v) NaCl) [1, 2]. Both *Gramella* strains utilize d-arabinose, l-arabinose, d-glucose and d-sucrose [1, 2], d-fructose and trehalose [8]. *G. portivictoriae* UST040801-001^T^ utilizes d-galactose, glycerol, d-mannitol, d-melibiose, d-sorbitol and starch [1], whereas *G. echinicola*JCM 13510^T^ utilizes d-xylose [7], but not d-lactose, d-mannose, d-mannitol, inositol, sorbitol, malonate and citrate [2]. A list of carbon sources utilized by both strains using the Biolog GN2 plate can be seen in Cho et al. [5].

#### Chemotaxonomic data

**Fig. 3:**
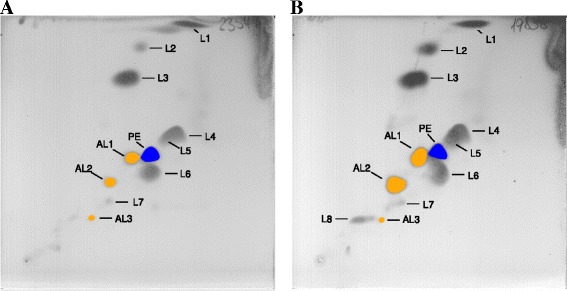
Polar lipids profiles of *G. echinicola* DSM 19838^T^ and *G. portivictoriae* DSM 23547^T^. The polar lipids were extracted using a modified method of Bligh and Dyer [55] (see Tindall [56]) and separated by two-dimensional thin-layer chromatography using the solvents chloroform/methanol/water (65:2:4, by vol.) in the first dimension and chloroform/methanol/acetic acid/water (80:12:15:4, by vol.) in the second dimension at 25 °C, as described by Tindall et al. [21]. For identification of the total polar lipids plates were sprayed with molybdatophosphoric acid (5 % in ethanol) and specific spray reagents used to detect the functional head groups of the lipids, as described by Tindall et al. [21]. PE, phosphatidylethanolamine (blue, phospholipid); AL, amino lipid (yellow, amino lipid); L, polar lipid

Major fatty acids (>5 % of total) of *G. echinicola*KMM 6050^T^ are C_15:0_, *anteiso*-C_15:0_, *iso*-C_15:0_, *iso*-C_16:0_, *iso*-C_16:1_, and *iso*-C_16:0_ 3-OH, *iso*-C_17:0_ 3-OH and summed feature 3 (*iso*-C_15:0_ 2-OH and/or C_16:1_ ω7c) [2]. Major fatty acids of *G. portivictoriae* UST040801-001^T^ are almost identical with the exception that C_15:0_ was not identified but *iso*-C_15:0_ 3-OH, *iso*-C_17:1_ ω9c [1]. The major polar lipids of strains DSM 19838^T^ and DSM 23547^T^ are phosphatidylethanolamine, five unidentified lipids (L1 – L2, L4 – L6) and two unidentified aminolipids (AL1 – AL2). One unidentified aminolipid (AL3) and three unidentified lipids (L2, L7 – L8) appeared as minor components (Fig. 3). As mentioned in the description of the genus *Gramella*, the major respiratory quinone in both strains is menachinone-6 whereas flexirubin-type pigments were not observed, only non-diffusible carotenoid pigments [2]. The DNA G + C content of the type strains was previously determined as 39.6 mol% of *G. echinicola*KMM 6050^T^ and 39.9 mol% of *G. portivictoriae* UST040801-001^T^ [1, 2].

#### Organic matter degradation

Both *Gramella* strains hydrolyze casein, gelatin, starch and Tweens 20, 40, 60 and 80 as well as esculin ferric citrate, but not agar, chitin or cellulose (CM-cellulose or filter paper) [1, 2, 6]. *G. echinicola* hydrolyzed DNA [2] whereas *G. portivictoriae* did not [1]. For strains KCTC 12278^T^ and KCTC 22434^T^ activity of acid phosphatase, alkaline phosphatase, naphthol-AS-BI-phosphohydrolase, esterase (C4), esterase lipase (C8), cystine arylamidase, leucine arylamidase, valine arylamidase and α-glucosidase, β-glucosidase were observed, but not the activity of β-glucuronidase, α-mannosidase, α-fucosidase, lipase (C14) and trypsin [5]. However, Shahina et al. [10] showed the activity of trypsin, α-chymotrypsin, α-glucosidase and *N*-acetyl-β-glucosaminidase for *G. echinicola*KCTC 12278^T^. Nedashkovskaya et al. [2] showed β-galactosidase activity for *G. echinicola*KMM 6050^T^ and Cho et al. [5] showed the α-galactosidase activity for *G. echinicola*KMM 12278^T^. Furthermore, *G. portivictoriae* UST040801-001^T^ was described with positive α-chymotrypsin, lipase (C14), α-galactosidase, α-glucosidase, β-glucosidase, trypsin and naphthol-AS-BI-phosphohydrolase activity and without *N*-acetyl-β-glucosaminidase, arginine dihydrolase, lysine decarboxylase, ornithine decarboxylase, tryptophan deaminase activity [1].

**Fig. 4:**
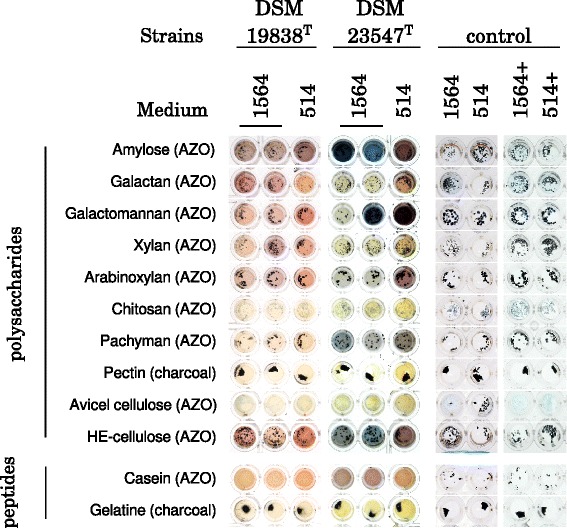
Polysaccharide hydrolysis by *Gramella* type strains *G. echinicola* DSM 19838^T^, *G. portivictoriae* DSM 23547^T^. Both strains were incubated in medium 514 (6 g/L carbon source mix) and HaHa (12 mg/L carbon source mix) for up to 14 days. *G. echinicola* DSM 19838^T^ was incubated at 25 °C and *G. portivictoriae* DSM 23547^T^ at 28 °C. Each 200 μL well of a microtiter plate was filled with a small portion of one of the AZO-CL-polysaccharides, −casein (Megazym, Bray, Ireland), charcoal-pectin, −gelatin in 100 μL medium. Each well was inoculated with 100 μL of a starved culture of the strains. The control wells were inoculated with 100 μL medium. The blue colour indicates the release of AZO- monomers and thus hydrolysis of the polysaccharide/peptide. A red-brown colour indicates growth of the strain (mixture of blue and yellow-orange). Black grains in the surrounding of the charcoal-pectin and -gelatine indicate hydrolysis

To get further insights into the polysaccharide decomposition potential of *G. echinicola*DSM 19838^T^ and *G. portivictoriae*DSM 23547^T^, both strains were incubated in HaHa medium (12 mg/L carbon source mix, [18]) and marine broth (6 g/L carbon source mix, DSMZ medium 514, [20]) supplemented with different polysaccharides, casein and gelatine at 25 °C for up to 14 days (Fig. 4). Each 200 μL well of a microtiter plate was filled with a small portion of one of the AZO-CL-polysaccharides, −casein (Megazym, Bray, Ireland), charcoal-pectin, −gelatin (chapter 15.3.32.3, method 3 in [21]) and 100 μL medium. Each well was inoculated with 100 μL of a starved culture or 100 μL medium as control. Both *Gramella* type strains hydrolyzed casein and starch but did not hydrolyze chitosan or cellulose (Avicel), as described in previous studies [1, 2, 6], galactomannan, arabinoxylan and hydroxyethyl-cellulose, but not pectin (Fig. 4). Pachyman was hydrolyzed by strain DSM 23547^T^, whereas galactan and xylan were hydrolyzed by strain DSM 19838^T^.

## Genome sequencing information

### Genome project history

*G. portivictoriae*DSM 23547^T^ and *G. echinicola*DSM 19838^T^ were selected for sequencing on the basis of their phylogenetic position [22] and are part of the *Genomic Encyclopedia of Type Strains*, Phase I: the one thousand microbial genomes project [23], a follow-up of the Genomic Encyclopedia of and *Archaea*: sequencing a myriad of type strains initiative [24] and the Genomic Standards Consortium project [25], which aim at increasing the number of key reference microbial genomes and to generate a large genomic basis for the discovery of genes encoding novel enzymes [26]. The genome project is deposited in the Genomes OnLine Database [27]. The permanent draft genome sequences are deposited in GenBank. Sequencing, finishing and annotation were performed by the DOE Joint Genome Institute [28]. A summary of the project information is shown in Table 2.

**Table 2:** Genome sequencing project information

MIGS ID	Property	Term	
		DSM 19838^T^	DSM 23547^T^
MIGS-31	Finishing quality	Level 2: Improved High-Quality Draft	Level 2: Improved High-Quality Draft
MIGS-28	Libraries used	Illumina Std shotgun library, 2 × 150bp	Illumina Std shotgun library, 2 × 150bp
MIGS-29	Sequencing platforms	Illumina HiSeq 2000	Illumina HiSeq 2000
MIGS-31.2	Fold coverage	123×	122×
MIGS-30	Assemblers	Velvet v. 1.1.04, ALLPATHS v. r41043	Velvet v. 1.1.04, ALLPATHS v. r41043
MIGS-32	Gene calling method	Prodigal, GenePRIMP, IMG-ER	Prodigal, GenePRIMP, IMG-ER
	Locus Tag	G530_RS01	G529_RS01
	NCBI project ID	16158	16157
	Genbank ID	AUHG00000000	AUHF00000000
	Genbank Date of Release	2015-08-15	2013-12-12
	GOLD ID	Gp0013656	Gp0013657
	BIOPROJECT	PRJNA185622	PRJNA185621
MIGS-13	Source Material Identifier	DSM 19838	DSM 23547
	Project relevance	Tree of Life, GEBA-KMG	Tree of Life, GEBA-KMG

### Growth conditions and genomic DNA preparation

Cultures of DSM 23547^T^ and DSM 19838^T^ were grown aerobically in DSMZ medium 514 [20] at 28 °C and 26 °C, respectively. Genomic DNA was isolated using Jetflex Genomic DNA Purification Kit (GENOMED 600100) following the standard protocol provided by the manufacturer but modified by an incubation time of 60 min, the incubation on ice overnight on a shaker, the use of an additional 50 μL proteinase K, and the addition of 200 μL protein precipitation buffer. DNA is available from the DSMZ through the DNA Bank Network [29].

### Genome sequencing and assembly

The draft genomes of DSM 19838^T^ and DSM 23547^T^ were generated using the Illumina technology [30]. An Illumina standard shotgun library was constructed and sequenced using the Illumina HiSeq 2000 platform which generated 13,321,360 reads totaling 1,998.2 Mb for strain DSM 19838^T^ and 9,930,650 reads totaling 1,489.6 Mb for strain DSM 23547^T^ (Table 3).

**Table 3:** Genome statistics

	DSM 19838^T^		DSM 23547^T^	
Attribute	Number	% of Total	Number	% of Total
Genome size (bp)	3,513,826	100.0	3,269,398	100.0
DNA coding (bp)	3,220,860	91.7	3,025,367	92.5
DNA G + C (bp)	1,296,572	36.9	1,292,347	39.5
DNA, scaffolds	18	100.0	8	100.0
Total genes	3,253	100.0	3,045	100.0
Protein coding genes	3,199	98.3	2,984	98.0
RNA genes	54	1.7	61	2.0
Pseudo genes	21	0.7	27	0.9
Genes in internal clusters	216	6.6	174	5.7
Genes with function prediction	2,464	75.8	2,302	75.6
Genes assigned to COGs	1,863	57.3	1,747	75.6
Genes with Pfam domains	2,564	78.8	2,409	79.1
Genes with signal peptides	334	10.3	347	11.4
Genes with transmembrane helices	766	23.6	662	21.7
CRISPR repeats	1	0.1	0	0.0

All general aspects of library construction and sequencing performed at the JGI can be found at the JGI website [31]. All raw sequence data were passed through DUK, a filtering program developed at JGI, which removes known Illumina sequencing and library preparation artifacts. The following steps were performed for assembly: filtered reads were assembled using Velvet [32], (2) 1–3 Kbp simulated paired end reads were created from Velvet contigs using wgsim [33], (3) sequence reads were assembled with simulated read pairs using Allpaths–LG [34]. Parameters for assembly steps were: (1) Velvet ("velveth 63 -shortPaired" and "velvetg -very clean yes -exportFiltered yes -min contig lgth 500 -scaffolding no -cov cutoff 10"), (2) wgsim ("wgsim -e 0–1 100–2 100 -r 0 -R 0 -X 0") (3) Allpaths–LG ("PrepareAllpathsInputs: PHRED 64 = 1 PLOIDY = 1 FRAG COVERAGE = 125 JUMP COVERAGE = 25 LONG JUMP COV = 50" and "RunAllpathsLG THREADS = 8 RUN = std shredpairs TARGETS = standard VAPI WARN ONLY = OVERWRITE = True").

The final draft assembly contained 18 contigs in a single scaffold for strain DSM 19838^T^ and 11 contigs in two scaffolds for strain DSM 23547^T^. The total size of the genome of strain DSM 19838^T^ is 3.5 Mbp and the final assembly is based on 430.3 Mbp of data, which provides a 122.6x average coverage of the genome. The total size of the genome of strain DSM 23547^T^ is 3.3 Mbp and the final assembly is based on 396.8 Mbp of data, which provides a 121.5x average coverage of the genome.

### Genome annotation

Genes were identified using Prodigal [35] as part of the DOE-JGI genome annotation pipeline [36], followed by manual curation using the JGI GenePRIMP pipeline [37]. The predicted CDSs were translated and used to search the National Center for Biotechnology Information non-redundant database, UniProt, TIGR-Fam, Pfam, PRIAM, KEGG, COG, and InterPro databases. The tRNAScanSE tool [38] was used to find tRNA genes, whereas ribosomal RNA genes were found by searches against models of the ribosomal RNA genes built from SILVA [39]. Other non-coding RNAs such as the RNA components of the protein secretion complex and the RNase P were identified by searching the genome for the corresponding Rfam profiles using INFERNAL [40]. Additional gene prediction analysis and manual functional annotation was performed within the Integrated Microbial Genomes-Expert Review platform [41] developed by the Joint Genome Institute, Walnut Creek, CA, USA [31]. CRISPRs were identified using the online CRIPSRFinder tool [42].

## Genome properties

The assemblies of the draft genome sequence of DSM 19838^T^ and DSM 23547^T^ consist of one and two scaffolds amounting to 3,513,826 bp and 3,269,398 bp, respectively (Table 3). The G + C content of DSM 19838^T^ is 36.9 %, which is 2.7 % less than the G + C content reported by Nedashkovskaya et al. [2], and thus shows a difference that surpasses the maximal range among strains belonging to the same species [43]. The G + C content of DSM 23547^T^ is 39.5 % and similar to the G + C content reported by Lau et al. [1]. From the genome of DSM 19838^T^ 3253 genes, 3199 protein-coding genes and 54 RNAs were predicted. From the genome of DSM 23547^T^ 3,045 genes, 2,984 protein-coding genes and 61 RNAs were predicted. The majority of the protein-coding genes (DSM 19838^T^, 75.8 %; DSM 23547^T^, 75.6 %) were assigned a putative function while the remaining ones were annotated as hypothetical proteins. The distribution of genes into COGs functional categories is presented in Table 4.

**Table 4:** Number of genes associated with the general COG functional categories

Code	DSM 19838^T^		DSM 23547^T^		Description
	Value	% age	Value	% age	
J	188	9.2	178	9.3	Translation, ribosomal structure and biogenesis
A	–	–	–	–	RNA processing and modification
K	108	5.3	99	5.2	Transcription
L	97	4.7	88	4.6	Replication, recombination and repair
B	1	0.1	1	0.1	Chromatin structure and dynamics
D	23	1.1	22	1.2	Cell cycle control, cell division, chromosome partitioning
V	63	3.1	54	2.8	Defense mechanisms
T	80	3.9	70	3.7	Signal transduction mechanisms
M	183	8.9	168	8.8	Cell wall/membrane biogenesis
N	15	0.7	19	1.0	Cell motility
U	21	1.0	19	1.0	Intracellular trafficking and secretion
O	102	5.0	91	4.8	Posttranslational modification, protein turnover, chaperones
C	101	4.9	107	5.6	Energy production and conversion
G	115	5.6	102	5.3	Carbohydrate transport and metabolism
E	182	8.9	188	9.8	Amino acid transport and metabolism
F	58	2.8	58	3.0	Nucleotide transport and metabolism
H	127	6.2	129	6.7	Coenzyme transport and metabolism
I	93	4.5	91	4.8	Lipid transport and metabolism
P	107	5.2	104	5.4	Inorganic ion transport and metabolism
Q	51	2.5	44	2.3	Secondary metabolites biosynthesis, transport and catabolism
R	218	10.6	189	9.9	General function prediction only
S	114	5.6	87	4.6	Function unknown
X	5	0.2	3	0.2	Mobilome: prophages, transposons
–	1,390	42.7	1,298	42.6	Not in COGs

## Insights from the genome sequence

### Comparative genomics

We present a brief comparative genomics analysis of *Gramella echinicola* and *Gramella portivictoriae* with a selection of its closest phylogenetic neighbors (according to Fig. 1), *'Gramella forsetii'* and *Zunongwangia profunda*. The genomes of these strains differ significantly in their size with 3.5 Mbp (*Gramella echinicola*), 3.3 Mbp (*Gramella portivictoriae*), 3.8 Mbp ('*Gramella forsetii*') and 5.1 Mbp (*Zunongwangia profunda*).

An estimate of the overall similarity among these four strains was generated with the Genome-to-Genome Distance Calculator (GGDC 2.0) [44, 45]. It calculates intergenomic distances by comparing two respective genomes to obtain HSPs (high- scoring segment pairs) and, then infers distances via a set of formulae (1, HSP length/total length; 2, identities/HSP length; 3, identities/total length). Formula 2 is robust against the use of incomplete genome sequences and the recommended choice [45]. For convenience the GGDC also reports model-based DDH estimates (digital DDH or dDDH) along with their confidence intervals [45].

The result of this comparison is shown in Table 5 and yields a dDDH value below 22 % throughout, i.e., clearly underlines the expected status of distinct species. With 21.3 % dDDH *Gramella echinicola* has the highest similarity to *'Gramella forsetii'*, whereas *Gramella portivictoriae* has the lowest similarity to *Zunongwangia profunda* with 18.2 % dDDH. The comparison of *Gramella echinicola* and *Gramella portivictoriae* yielded 18.4 % dDDH.

**Table 5:** Pairwise comparison of *Gramella echinicola* and *Gramella portivictoriae* with *'Gramella forsetii'* and *Zunongwangia profunda* using the GGDC 2.0 (Genome-to-Genome Distance Calculator). Digital DDH (dDDH) and the respective confidence intervals (C.I.) are specified for GGDC’s recommended formula 2

Strain 1	Strain 2	% dDDH	% C.I.
*G. echinicola* DSM 19838^T^	*’G. forsetii’* KT0803	21.3	2.3
*’G. forsetii’* KT0803	*G. portivictoriae* DSM 23547^T^	18.6	2.3
*G. echinicola* DSM 19838^T^	*G. portivictoriae* DSM 23547^T^	18.4	2.3
*’G. forsetii’* KT0803	*Zunongwangia profunda *SM A87^T^	20.4	2.3
*G. echinicola* DSM 19838^T^	*Zunongwangia profunda *SM A87^T^	18.6	2.3
*G. portivictoriae *DSM 23547^T^	*Zunongwangia profunda *SM A87^T^	18.2	2.3

### Gliding motility

As given in the description of the genus, all *Gramella* are motile by gliding [2]. We identified all of the genes in the genomes of both type strains that are essential for gliding- motility (Table 6). Furthermore, we observed different modes of gliding-motility on marine soft agar (medium 514 with 0.3 % agar) for both strains. Interestingly, the observed modes of gliding-motility corroborate the observed cellular morphologies (Fig. 2). *G. echinicola*DSM 19838^T^ moved by gliding with smooth and entire translucent margins and produced extracellular polymeric substances. In contrast, *G. portivictoriae*DSM 23547^T^ formed micro-colonies surrounding the original colony and produced appendages at the cell surface (Fig. 2).

**Table 6:** Gliding motility-related genes in strain DSM 19838^T^ and DSM 23547^T^ compared to genes in *F. johnsoniae* studied by McBride and Zhu [72]

		*G. echinicola* 19838^T^	*G. portivictoriae* 23547^T^	*F. johnsoniae* ATCC 17061^T^
Locus tag prefix		G530_RS01	G529_RS01	FJOH_
Gliding motility		+	+	+
Adhesin-like				
	*remA*	–	–	0808
	*remB*	04710	03110	1657
	*sprB*	00190	–	0979
ATP-binding cassette transporter				
	*gldA*	13745	03925	1516
	*gldF*	00125	12395	2722
	*gldG*	00120	12390	2721
Additional proteins				
	*gldB* ^a^	05595	08905	1793
	*gldC*	05600	08910	1794
	*gldD* ^a^	03500	02145	1540
	*gldE*	03505	02150	1539
	*gldH* ^a^	01530	00125	0890
	*gldJ* ^a^	05045	08395	1557
peptidoprolyl isomerase (*Flavobacteriia*, protein folding)				
	*gldI*	12360	06845	2369
Type IX secretion system (secretion of RemA/RemB)				
	*gldK* ^a^	14425	05780	1853
	*gldL* ^a^	14430	05775	1854
	*gldM* ^a^	14435	05770	1855
	*gldN* ^a^	14440	05765	1856, 1857
	*sprA* ^a^	04685	03085	1653
	*sprE* ^a^	01675	00280	1051
	*sprT* ^a^	15350	04170	1466

^a^essential gliding motility genes after McBride and Zhu [72]

### Peptidases

The MEROPS [46] annotation was carried out by searching the sequences against MEROPS 9.10 (access date: 2014.10.16, version: pepunit.lib) as described by Hahnke et al. [15]. *G. echinicola*DSM 19838^T^ processes 161 peptidases the majority of which were 68 metallo (M) and 62 serine (S) peptidases (Table 7 and Table S1 in Additional file 1). Furthermore, the genome contained 17 simple peptidase inhibitors (Table 7 and Table S2 in Additional file 1). *G. portivictoriae*DSM 23547^T^ processes 181 peptidases the majority of which were 81 metallo (M) and 72 serine (S) peptidases (Table 7 and Table S3 in Additional file 1). The genome contained 21 simple peptidase inhibitors (Table 7 and Table S4 in Additional file 1).

**Table 7:** Peptidases and simple peptidase inhibitors in the genome of strains DSM 19838^T^ and DSM 23547^T^

Peptidase	Number of genes	
family	DSM 19838^T^	DSM 23547^T^
M01	5	4
M03	2	2
M12	2	2
M13	1	1
M14	6	7
M15	1	1
M16	6	5
M19	1	1
M20	6	5
M23	8	10
M24	4	4
M28	6	5
M38	12	6
M41	1	1
M42	1	1
M43	2	1
M48	2	3
M49	2	0
M50	1	1
M56	3	1
M57	1	1
M61	2	2
M75	1	1
M79	3	1
M97	2	2
A08	1	1
A28	1	1
S01	1	2
S06	0	1
S08	2	3
S09	22	19
S10	1	1
S12	9	4
S13	1	1
S14	2	2
S15	1	0
S16	3	3
S24	1	2
S26	1	1
S33	15	13
S41	6	4
S41	6	4
S51	1	1
S54	4	4
S66	1	1
N11	0	1
C01	1	0
C26	6	6
C40	4	4
C44	5	5
C45	1	1
C56	4	4
C82	1	1
T02	2	2
T03	0	1
U32	2	2
I4	1	1
I39	18	15
I43	1	0
I87	1	1

### Carbohydrate active enzymes

*G. echinicola*DSM 19838^T^ and *G. portivictoriae*DSM 23547^T^ harboured a large set of 127 and 119 CAZymes, respectively, comprising 37–39 glycoside hydrolases, 2–5 polysaccharide lyases, 9–14 carbohydrate esterases, 9–10 carbohydrate binding modules and 55–61 glycoside transferases (Table 8 and Table S5 and S6 in Additional file 1).

**Table 8:** Carbohydrate active enzymes (CAZy) in the genome of strains DSM 19838^T^ and DSM 23547^T^

CAZy	Number of genes	
family	DSM 19838^T^	DSM 23547^T^
GH2	1	2
GH3	4	3
GH5	1	3
GH9	0	1
GH13	6	2
GH15	1	1
GH16	5	3
GH17	1	1
GH20	1	0
GH23	2	2
GH26	1	2
GH27	0	1
GH28	1	0
GH29	1	0
GH31	1	1
GH32	1	4
GH37	1	0
GH43	2	1
GH63	0	1
GH65	0	1
GH73	1	1
GH88	1	0
GH97	1	1
GH105	1	0
GH113	1	1
GH130	0	1
GH^a^	1	3
CE1	1	0
CE4	3	1
CE8	1	0
CE11	1	1
CE12	1	0
CE14	3	2
CE^a^	4	2
PL6	0	1
PL7	0	2
PL9	1	0
PL12	0	1
PL17	0	1
CBM38	0	1
CBM48	2	2
CBM50	4	4
CBM57	2	1
CBM^a^	2	1
GT2	29	26
GT4	18	16
GT5	2	1
GT8	0	1
GT9	2	1
GT10	2	0
GT19	1	1
GT20	1	1
GT28	1	1
GT30	1	1
GT51	3	3
GT83	1	1
GT^a^	0	2
AA1	1	0
AA6	0	1
AA12	1	0
AA^a^	0	2

^a^genes attributed to an enzyme class, but not to a family

### Polysaccharide utilization loci

**Fig. 5:**
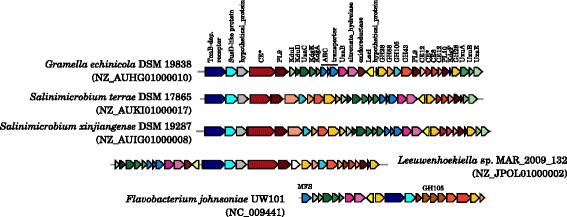
A pectin-like PUL of *G. echinicola* DSM 19838^T^ and other *Flavobacteriaceae*. A similar PUL was identified in *Flavobacterium johnsoniae* UW101^T^ by McBride et al. [16]. Locus tags are given below both the first and last gene of the loci. Accession numbers in brackets are GenBank accession numbers of the corresponding contig. Investigation of syntenic loci was done using MultiGeneBlast [57]. A description of glycoside hydrolase (GH), polysaccharide lyase (PL) and carbohydrate esterase (CE) families can be seen at the CAZy homepage [58, 59]. The pectin-like polysaccharide decomposition pathway, encoded by these genes, is shown in Figure S6 in the Additional file 2. SusD, SusD-like protein; LacI, LacI family transcriptional regulator; MFS, major facilitator superfamily transporter; KduD, 2-keto-3-deoxy-d-gluconate-dehydrogenase; UxaB, altronate oxidoreductase; UxaC, glucuronate isomerase; KdgA, 2-keto-3-deoxygluconate-6-phosphate aldolase; KdgF, pectin degradation protein; KduI, 5-dehydro-4-deoxy- d-glucuronate isomerase; KdgK, 2-dehydro-3-deoxygluconokinase; UxuA, mannonate dehydratase; UxuB, d-mannonate oxidoreductase; UxaE, d-tagaturonate epimerase

Kabisch et al. [13] investigated ’*G. forsetii*’ KT0803 for its ability to decompose laminarin-like, α-1,4-linked-glucose and alginate-like polysaccharides. The two PULs involved in either the decomposition of laminarin-like polysaccharides or α-1,4-linked glucose-polymers (glycogen, starch and amylose) were as well found in *G. portivictoriae*DSM 23547^T^ and *G. echinicola*DSM 19838^T^ (Figure S1, Figure S2 in Additional file 2). Both PULs were greatly conserved among other closely related genera (see Fig. 1) and within the *Flavobacteriaceae*. The PUL involved in the decomposition of alginate-like polysaccharides was found in *G. portivictoriae*DSM 23547^T^, but not in *G. echinicola*DSM 19838^T^ (Figure S3 and Figure S4 in Additional file 2). This PUL was not conserved among other closely related genera, but greatly distributed within the *Flavobacteriaceae*. Interestingly, the PULs of the *Salegentibacter* and *Aquimarina* were highly syntenic with those of *Gramella*, whereas the PULs of *Gillisia*, *Mesonia*, *Zunongwangia*, *Psychroflexus*, *Salinimicrobium* and *Dokdonia* had additional genes. One PUL that potentially encodes for the decomposition of sulfated β-d-glucosides (Figure S5 in Additional file 2) and one for the decomposition of β-d-fructans (levans) (Figure S6 in Additional file 2) was found in all three *Gramella* and in other closely related *Flavobacteriaceae*. A PUL that was found only in *G. echinicola*DSM 19838^T^ comprised pectin-like polysaccharide decomposing CAZymes and genes of the pectate degradation pathway (Fig. 5, Figure S7 in Additional file 2). A similar set of genes was found in a PUL of *Flavobacterium johnsoniae* UW101^T^, which was hypothesized to be involved in pectin decomposition [16].

**Fig. 6:**
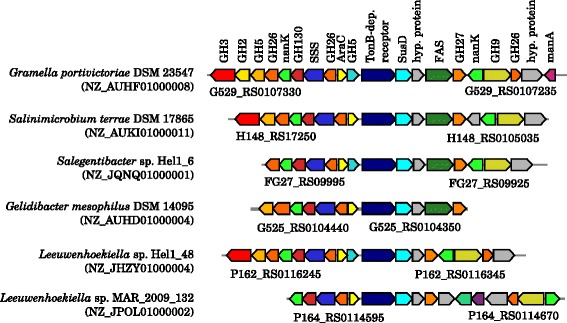
A cellulose/hemicellulose-like PUL of *G. portivictoriae* DSM 23547^T^ and other *Flavobacteriaceae*. Locus tags are given below both the first and last gene of the loci. Accession numbers in brackets are GenBank accession numbers of the corresponding contig. Investigation of syntenic loci was done using MultiGeneBlast [57]. A description of glycoside hydrolase (GH), polysaccharide lyase (PL) and carbohydrate esterase (CE) families can be seen at the CAZy homepage [58, 59]. SusD, SusD-like protein; AraC, AraC family transcriptional regulator; manA, Man-6-P isomerase; nanK, GlcNAc-2-epimerase; FAS, FAS1 domain protein; SSS, sodium:solute symporter

Surprisingly, we found a PUL in *G. portivictoriae*DSM 23547^T^, *Salinimicrobium terrae*DSM 17865^T^ and some other *Flavobacteriaceae* (Fig. 6) comprising typical cellulases/hemicellulases, such as GH5 (cellulase family A), GH9 (cellulase family E) and GH26 (cellulase family I). However, *Salinimicrobium terrae*DSM 17865^T^ was described to be unable to hydrolyze carboxymethyl-cellulose and filter paper. Lau et al. [1] showed β-glucosidase activity by *G. portivictoriae*DSM 23547^T^, but no decomposition of carboxymethyl-cellulose. The authors tested cellulose decomposition using a 0.5 % CMC overlay agar as described by McCammon et al. [47]. As mentioned above, we could show that *G. portivictoriae*DSM 23547^T^ is able to hydrolyze hydroxyethyl-cellulose, but not Avicel-cellulose. Thus we additionally tested this strain for the decomposition of AZO-CL carboxymethyl-cellulose, Whatman filter No. 1 cellulose and cellulose of cigarette paper. In HaHa medium and marine broth strain DSM 23547^T^ hydrolyzed AZO-CL carboxymethyl-cellulose, but not the Whatman filter.

## Conclusion

All three of the genome-sequenced *Gramella* spp. sequenced to date were isolated from marine habitats, *Gramella echinicola*DSM 19838^T^ was isolated from a sea urchin, *G. portivictoriae*DSM 23547^T^ from the sediment and ’*G. forsetii*’ KT0803 from surface seawater. In contrast to ’*G. forsetii*’ (48.7 peptidases Mbp^−1^) [14, 48], both *G. echinicola*DSM 19838^T^ and *G. portivictoriae* DSM 23547^T^ have a greater number peptidases, 68 Mbp^−1^ and 81 Mbp^−1^, respectively. The observed dominance of metallo (M), serine (S) and cysteine (C) peptidase families was already reported by Xing and Hahnke et al. [14] and seems to be a general feature among *Flavobacteriaceae*. Interestingly, while both *G. echinicola*DSM 19838^T^ and *G. portivictoriae* DSM 23547^T^ have a similar amount of CAZymes (119 and 127), CAZymes Mbp^−1^ (36.1 and 36.4) and CAZy families (44 and 45), the genome of ’*G. forsetii*’ comprised a larger amount of CAZymes (164 overall and 43.2 Mbp^−1^) and a greater diversity of CAZy families (54) [13, 14]. We observed different polysaccharide decomposition capabilities among the *Gramella* which might be linked to the nutrient composition of the habitats they were isolated from. Whether the laminarin-like and the starch/amylose-like PUL is a common feature of *Gramella* needs to be assessed once further *Gramella* genomes are available. Furthermore, the link between the coincidence of the observed gliding-motility modes, the cellular morphologies and certain environmental conditions has to be investigated in detail. For example, *Gramella oceani* and *Muricauda ruestringensis*, both producing appendages, were isolated from marine intertidal sediment [6, 49]. Bruns et al. [49] and Hahnke et al. [50] assumed that such appendages are connections between the cells or serve as anchor to mediate surface attachment and particle formation.

## Taxonomic and nomenclatural proposals

Based on the new morphological (gliding, EPS, appendages), physiological (polysaccharide hydrolysis) and genomic observations (DNA G + C content, CAZymes, PUL, peptidases) we propose the emendation of *Gramella echinicola*DSM 19838^T^ Nedashkovskaya et al. [2] emend. Shahina et al. [10] and the emendation of *Gramella portivictoriae* Lau et al. [5].

### Emended description of *Gramella echinicola* Nedashkovskaya et al. [2] emend. Shahina et al. [10]

The description of *Gramella echinicola* is as given by Nedashkovskaya et al. [2] and Shahina et al. [10], with the following emendations. The major polar lipids are phosphatidylethanolamine, together with a number of unidentified lipids, that included seven polar lipids that did not stain with any of the specific spray reagents (L1 – L8) and two amino lipids (AL1 – AL3) that together with their specific Rf values, that can be deduced from Fig. 3 and their staining behavior, may serve as reference points for future work where chromatographic conditions are the same. The G + C content is 36.9 %. Production of extracellular polymeric substances. Hydrolyses aesculin, galactomannan, arabinoxylan, galactan, xylan and hydroxyethyl-cellulose, but not Avicel-cellulose, pectin and chitosan.

### Emended description of *Gramella portivictoriae* Lau et al. [1]

The description of *Gramella portivictoriae* is as given by Lau et al. [1], with the following emendations. The major polar lipids are phosphatidylethanolamine, together with a number of unidentified lipids, that included seven polar lipids that did not stain with any of the specific spray reagents (L1 – L7) and two amino lipids (AL1 – AL3) that together with their specific Rf values, that can be deduced from Fig. 3 and their staining behavior, may serve as reference points for future work where chromatographic conditions are the same. Appendages at the cell surface. Hydrolyses aesculin, galactomannan, arabinoxylan, pachyman and hydroxyethyl-cellulose, but not Avicel-cellulose, pectin and chitosan.

## Additional files

Additional file 1: Table S1. Peptidases or homologues in the genome of *Gramella echinicola* DSM 19838^T^. **Table S2.** Simple peptidases inhibitors in the genome of *Gramella echinicola* DSM 19838^T^. **Table S3.** Peptidases or homologues in the genome of *Gramella portivictoriae* DSM 23547^T^. **Table S4.** Simple peptidases inhibitors in the genome of *Gramella portivictoriae* DSM 23547^T^. **Table S5.** Carbohydrate active enzymes (CAZymes) in the genome of *Gramella echinicola* DSM 19838^T^. **Table S6.** Carbohydrate active enzymes (CAZymes) in the genome of *Gramella portivictoriae* DSM 23547^T^. (PDF 261 kb) Additional file 2: Figure S1. The laminarin-like PUL. **Figure S2.** The 1,4-linked glucose-polymer-like PUL. **Figure S3.** The alginate-like PUL. **Figure S4.** Part of the alginate-like polysaccharide decom- position pathway. **Figure S5.** The sulfated β-d-glucoside PUL. **Figure S6.** The two combined β-d-fructans PUL. **Figure S7.** Part of the pectin-like polysaccharide decomposition pathway. (PDF 642 kb)

## Abbreviations

AZO-CL: Azurine-crosslinked
CAZy: Carbohydrate active enzymes
EPS: Extracellular polymeric substances
PUL: Polysaccharide utilization loci
