# Supplementary material for: Comparing polysaccharide decomposition between the type strains Gramella echinicola KMM 6050T (DSM 19838T) and Gramella portivictoriae UST040801-001T (DSM 23547T), and emended description of Gramella echinicola Nedashkovskaya et al. 2005 emend. Shahina et al. 2014 and Gramella portivictoriae Lau et al. 2005
Source: Stand Genomic Sci. 2016 Jun 3;11:37. doi: 10.1186/s40793-016-0163-9 (PMC4891872; doi:10.1186/s40793-016-0163-9)
Supplement: Additional file 1: Table S1. — Peptidases or homologues in the genome of Gramella echinicola DSM 19838T. Table S2. Simple peptidases inhibitors in the genome of Gramella echinicola DSM 19838T. Table S3. Peptidases or homologues in the genome of Gramella portivictoriae DSM 23547T. Table S4. Simple peptidases inhibitors in the genome of Gramella portivictoriae DSM 23547T. Table S5. Carbohydrate active enzymes (CAZymes) in the genome of Gramella echinicola DSM 19838T. Table S6. Carbohydrate active enzymes (CAZymes) in the genome of Gramella portivictoriae DSM 23547T. (PDF 261 kb) [file 40793_2016_163_MOESM1_ESM.pdf]

Comparing polysaccharide decomposition among the type strains *Gramella echinicola* KMM 6050^{T} (DSM 19838^{T}) and *Gramella portivictoriae* UST040801-001^{T} (DSM 23547^{T}), and emended description of *Gramella echinicola* Nedashkovskaya et al. 2005 emend. Shahina et al. 2014 and *Gramella portivictoriae* Lau et al. 2005

Irina Panschin[1], Sixing Huang[1], Jan P. Meier-Kolthoff[1], Brian J. Tindall[1], Manfred Rohde[2], Alla Lapidus[3], James Han[4], Stephan Trong[4], Matthew Haynes[4], T.B.K. Reddy[4], Marcel Huntemann[4], Amrita Pati[4], Natalia N. Ivanova[4], Konstantinos Mavromatisv[5], Victor Markowitz[5], Tanja Woyke[4], Markus Göker[1], Hans-Peter Klenk[6], Nikos C. Kyrpides[5,7] and Richard L. Hahnke[1,*]

[1] Leibniz Institute DSMZ – German Collection of Microorganisms and Cell Cultures, Braunschweig, Germany. [2] Helmholtz Centre for Infection Research, Braunschweig, Germany. [3] Algorithmic Biology Lab, St. Petersburg Academic University, St.Petersburg, Russia. [4] Genome Biology Program, Department of Energy Joint Genome Institute, Walnut Creek, California, USA. [5] Biological Data Management and Technology Center, Lawrence Berkeley National Laboratory, Berkeley, California, USA. [6] School of Biology, Newcastle University, Newcastle upon Tyne, UK. [7] School of Biology, King Abdulaziz University, Jeddah, Saudi Arabia.
*Corresponding author: Richard L. Hahnke, richard.hahnke@dsmz.de

Additional file 1 – Supporting Information – Tables

Table S1: Peptidases or homologues in the genome of *Gramella echinicola* DSM 19838^{T}.

Accession No.	family	Peptidase or homologue
WP_026932724.1	M28X	family M28 unassigned peptidases (Gramella forsetii)
WP_026932733.1	M43B	ulilysin (Gramella forsetii)
WP_026932742.1	S33	SCO7095-type peptidase (Gramella forsetii)
WP_026932743.1	M24B	subfamily M24B unassigned peptidases (Gramella forsetii)
WP_026932773.1	M14X	family M14 non-peptidase homologues (Croceibacter atlanticus)
WP_026932777.1	A08	signal peptidase II (Gramella forsetii)
WP_026932804.1	M50B	subfamily M50B unassigned peptidases (Gramella forsetii)
WP_026932839.1	S12	family S12 unassigned peptidases (Zunongwangia profunda)
WP_026932851.1	U32	family U32 unassigned peptidases (Elizabethkingia anophelis)
WP_026932891.1	M23B	subfamily M23B unassigned peptidases (Gramella forsetii)
WP_026932916.1	S41A	CtpC peptidase (Zobellia galactanivorans)
WP_026932918.1	M01	family M1 unassigned peptidases (Gramella forsetii)
WP_026932919.1	S08A	subfamily S8A unassigned peptidases (Zunongwangia profunda)
WP_026932932.1	C40	family C40 unassigned peptidases (Gramella forsetii)
WP_026932938.1	S12	family S12 unassigned peptidases (Zunongwangia profunda)
WP_026932956.1	S09A	prolyl oligopeptidase (Gramella forsetii)
WP_026932972.1	S09X	family S9 unassigned peptidases (Gramella forsetii)
WP_026932975.1	U32	family U32 unassigned peptidases (Chthonomonas calidirosea)
WP_026932978.1	C44	family C44 unassigned peptidases (Gramella forsetii)
WP_026932996.1	T02	family T2 unassigned peptidases (Gramella forsetii)
WP_026933028.1	S33	family S33 unassigned peptidases (Gramella forsetii)
WP_026933039.1	S09B	subfamily S9B unassigned peptidases (Algoriphagus sp. PR1)
WP_026933040.1	M28D	subfamily M28D unassigned peptidases (Cellulophaga algicola)
WP_026933047.1	I39	family I39 unassigned peptidase inhibitor homologues (Pedobacter heparinus)
WP_026933078.1	S12	family S12 unassigned peptidases
WP_026933102.1	S41A	CtpC peptidase (Lacinutrix sp. 5H-3-7-4)
WP_026933117.1	S12	family S12 unassigned peptidases (Marivirga tractuosa)
WP_026933119.1	C56	family C56 non-peptidase homologues (Gramella forsetii)
WP_026933125.1	S33	family S33 unassigned peptidases (Gramella forsetii)
WP_026933171.1	M23B	Mername-AA292 peptidase (Gramella forsetii)
WP_026933172.1	S16	DNA repair protein RadA (Escherichia coli) (Croceibacter atlanticus)
WP_026933179.1	C44	glucosamine-fructose-6-phosphate aminotransferase
WP_026933183.1	M61	family M61 unassigned peptidases (Gramella forsetii)
WP_026933214.1	M16B	subfamily M16B non-peptidase homologues (Robiginitalea biformata)
WP_026933278.1	S09X	family S9 non-peptidase homologues
WP_026933290.1	S33	family S33 unassigned peptidases (Carnobacterium sp. WN1359)
WP_026933294.1	M79	family M79 unassigned peptidases (Gramella forsetii)
WP_026933298.1	S12	family S12 unassigned peptidases (Cellulophaga lytica)
WP_026933306.1	M24B	aminopeptidase P (bacteria) (Gramella forsetii)
WP_026933311.1	S33	family S33 non-peptidase homologues (Microscilla marina)
WP_026933312.1	M20A	subfamily M20A unassigned peptidases (Fulvivirga imtechensis)

Continued on next page

Table S1: (continued)

Accession No.	family	Peptidase or homologue
WP_026933317.1	M20F	subfamily M20F unassigned peptidases (Lacinutrix sp. 5H-3-7-4)
WP_026933337.1	M56	family M56 unassigned peptidases (Gramella forsetii)
WP_026933365.1	M38	family M38 non-peptidase homologues (Gramella forsetii)
WP_026933394.1	T02	Chryseobacterium meningosepticum-type N4-(beta-N-acetylglucosaminyl)-L-asparaginase (Gramella forsetii)
WP_026933400.1	S33	family S33 unassigned peptidases (Pseudomonas mendocina)
WP_026933464.1	S33	family S33 non-peptidase homologues (Gramella forsetii)
WP_026933469.1	S14	peptidase Clp (type 1) (Gramella forsetii)
WP_026933476.1	M23B	Mername-AA292 peptidase (Gramella forsetii)
WP_026933502.1	M28E	subfamily M28E unassigned peptidases (Gramella forsetii)
WP_026933517.1	M03A	subfamily M3A unassigned peptidases (Gramella forsetii)
WP_026933566.1	C26	family C26 non-peptidase homologues (Gramella forsetii)
WP_026933597.1	S33	family S33 unassigned peptidases (Gramella forsetii)
WP_026933616.1	S66	family S66 unassigned peptidases (Gramella forsetii)
WP_026933621.1	C44	AsnB protein (Gramella forsetii)
WP_026933639.1	S51	alpha-aspartyl dipeptidase (eukaryote) (Gramella forsetii)
WP_026933644.1	M01	family M1 unassigned peptidases (Gramella forsetii)
WP_026933677.1	M41	Afg3-like protein 2 (Gramella forsetii)
WP_026933683.1	M14X	family M14 non-peptidase homologues (Polaribacter sp. MED152)
WP_026933697.1	C26	TRP3 protein
WP_026933711.1	C56	family C56 non-peptidase homologues (Spirosoma linguale)
WP_026933763.1	M38	family M38 non-peptidase homologues (Gramella forsetii)
WP_026933764.1	M38	family M38 non-peptidase homologues (Spirosoma linguale)
WP_026933770.1	M14X	family M14 non-peptidase homologues (Gramella forsetii)
WP_026933778.1	M20B	peptidase T (Gillisia limnaea)
WP_026933868.1	S33	family S33 non-peptidase homologues (Gramella forsetii)
WP_026933879.1	S09D	glutamyl endopeptidase C (Gramella forsetii)
WP_026933895.1	M20D	carboxypeptidase Ss1 (Gillisia limnaea)
WP_026933899.1	S33	CPO-A2 (Streptomyces aureofaciens)-type chloroperoxidase (Gramella forsetii)
WP_026933908.1	M79	family M79 unassigned peptidases (Gramella forsetii)
WP_026933911.1	M24X	family M24 non-peptidase homologues (Leeuwenhoekiella blandensis)
WP_026933913.1	M01	family M1 unassigned peptidases (Zunongwangia profunda)
WP_026933973.1	M16B	subfamily M16B non-peptidase homologues (Gramella forsetii)
WP_026933974.1	M14X	family M14 non-peptidase homologues (Gramella forsetii)
WP_026933979.1	C26	GMP synthase (Gillisia limnaea)
WP_026933980.1	C40	spr peptidase (Thermodesulfobacterium sp. OPB45)
WP_026933984.1	S08A	subfamily S8A unassigned peptidases (Gramella forsetii)
WP_026933990.1	C26	CTP synthetase (Zunongwangia profunda)
WP_026934002.1	S09C	dpf-6 g.p. (Caenorhabditis elegans) (Gramella forsetii)
WP_026934004.1	S09X	family S9 non-peptidase homologues (Gramella forsetii)
WP_026934005.1	S09C	subfamily S9C unassigned peptidases
WP_026934014.1	C82	family C82 unassigned peptidases (Chlorobium phaeobacteroides)
WP_026934037.1	S41A	subfamily S41A non-peptidase homologues
WP_026934046.1	S15	family S15 unassigned peptidases (Croceibacter atlanticus)

Continued on next page

Table S1: (continued)

Accession No.	family	Peptidase or homologue
WP_026934070.1	S16	family S16 non-peptidase homologues (Croceibacter atlanticus)
WP_026934094.1	M20A	BT3549 putative peptidase (Zobellia galactanivorans)
WP_026934129.1	M12A	vertebrate tolloid-like 1 protein (Gramella forsetii)
WP_026934130.1	M12A	blastula protease 10 (Paracentrotus) (Gramella forsetii)
WP_026934134.1	S54	family S54 unassigned peptidases (Gramella forsetii)
WP_026934195.1	S10	family S10 unassigned peptidases (Gramella forsetii)
WP_026934198.1	M61	family M61 unassigned peptidases (Gramella forsetii)
WP_026934208.1	M03A	subfamily M3A unassigned peptidases (Gramella forsetii)
WP_026934216.1	M49	family M49 unassigned peptidases (Pseudoalteromonas atlantica)
WP_026934316.1	C45	family C45 unassigned peptidases (Flavobacterium johnsoniae)
WP_026934334.1	S12	family S12 unassigned peptidases (Gramella forsetii)
WP_026934379.1	M16B	subfamily M16B non-peptidase homologues (Bacteroides sp. CF50)
WP_026934415.1	M19	family M19 unassigned peptidases (Gramella forsetii)
WP_026934444.1	C40	family C40 unassigned peptidases (Gramella forsetii)
WP_026934459.1	C56	PfpI peptidase (Gramella forsetii)
WP_026934464.1	M14X	family M14 non-peptidase homologues (Gramella forsetii)
WP_026934494.1	M38	family M38 non-peptidase homologues (Zunongwangia profunda)
WP_026934520.1	M16X	family M16 unassigned peptidases (Imtechella halotolerans)
WP_026934596.1	S41A	subfamily S41A unassigned peptidases (Croceibacter atlanticus)
WP_026934606.1	C26	dihydro-orotase (N-terminal unit) (Homo sapiens-type) (Ailuropoda melanoleuca)
WP_026934627.1	C56	KIAA0361 protein (Homo sapiens-type) (Gramella forsetii)
WP_026934634.1	M43B	ulilysin (Gramella forsetii)
WP_026934639.1	M28E	subfamily M28E non-peptidase homologues (Gramella forsetii)
WP_026934655.1	S09X	family S9 unassigned peptidases (Gramella forsetii)
WP_026934725.1	S01C	DegQ peptidase (Krokinobacter sp. 4H-3-7-5)
WP_026934743.1	M15D	vanX D-Ala-D-Ala dipeptidase (Gramella forsetii)
WP_026934770.1	M28A	subfamily M28A unassigned peptidases (Psychroflexus torquis)
WP_026934811.1	M38	1300019j08rik protein (Gramella forsetii)
WP_026934830.1	M48A	zmpste24 g.p. (Dictyostelium discoideum-type) (Gramella forsetii)
WP_026934832.1	M38	family M38 non-peptidase homologues (Gramella forsetii)
WP_026934833.1	M38	family M38 non-peptidase homologues (Gramella forsetii)
WP_026934835.1	M20C	Pep581 peptidase (Gillisia sp. CBA3202)
WP_026934849.1	S54	RhoII peptidase (Haloferax volcanii) and similar (Gramella forsetii)
WP_026934850.1	S54	family S54 unassigned peptidases (Gramella forsetii)
WP_026934853.1	S26A	subfamily S26A unassigned peptidases (Gramella forsetii)
WP_026934867.1	S33	family S33 unassigned peptidases (Stigmatella aurantiaca)
WP_026934903.1	M38	family M38 unassigned peptidases (Dyadobacter fermentans)
WP_026934944.1	M97	family M97 unassigned peptidases (Pontibacter sp. BAB1700)
WP_026934948.1	S09A	oligopeptidase B (Gramella forsetii)
WP_026934981.1	C26	imidazole glycerol phosphate synthase subunit HisH (Gramella forsetii)

Continued on next page

Table S1: (continued)

Accession No.	family	Peptidase or homologue
WP_026934993.1	S09B	subfamily S9B unassigned peptidases (Haliscomenobacter hydrossis)
WP_026935027.1	S09X	family S9 non-peptidase homologues (Spirosoma linguale)
WP_026935033.1	M38	family M38 non-peptidase homologues (Sphingomonas wittichii)
WP_026935038.1	S12	family S12 unassigned peptidases (Chryseobacterium meningosepticum)
WP_026935041.1	M01	family M1 unassigned peptidases (Psychroflexus torquis)
WP_026935046.1	S09X	family S9 non-peptidase homologues (Bacteroides sp. 3 1 33FAA)
WP_026935048.1	S12	family S12 unassigned peptidases (Lacinutrix sp. 5H-3-7-4)
WP_026935051.1	S09X	family S9 non-peptidase homologues (Kordia algicida)
WP_026935056.1	S09X	family S9 non-peptidase homologues (Cellulophaga algicola)
WP_026935060.1	S09B	subfamily S9B unassigned peptidases (Muricauda ruestringensis)
WP_026935101.1	S33	family S33 unassigned peptidases (Gramella forsetii)
WP_026935145.1	S24	family S24 unassigned peptidases (Zunongwangia profunda)
WP_026935178.1	S09X	family S9 unassigned peptidases (Muricauda ruestringensis)
WP_026935263.1	S09B	dipeptidyl-peptidase 4 (bacteria-type 2) (Lacinutrix sp. 5H-3-7-4)
WP_026935295.1	M97	family M97 unassigned peptidases (Gramella forsetii)
WP_026935316.1	S09X	family S9 non-peptidase homologues (Gramella forsetii)
WP_026935321.1	S16	Lon-A peptidase (Gramella forsetii)
WP_026935327.1	M48C	subfamily M48C unassigned peptidases (Gramella forsetii)
WP_026935328.1	S09X	family S9 unassigned peptidases (Gramella forsetii)
WP_026935339.1	M23B	subfamily M23B unassigned peptidases (Polaribacter sp. MED152)
WP_026935342.1	M23B	subfamily M23B unassigned peptidases (Gramella forsetii)
WP_026935394.1	M24A	methionyl aminopeptidase 1 (Escherichia-type) (Gramella forsetii)
WP_026935400.1	S14	peptidase Clp (type 1) (Gramella forsetii)
WP_026935415.1	M57	prtB g.p. (Myxococcus xanthus) (Gillisia limnaea)
WP_026935424.1	M56	family M56 unassigned peptidases (Gramella forsetii)
WP_026935473.1	M23B	subfamily M23B unassigned peptidases (Gramella forsetii)
WP_026935510.1	C44	AsnB protein (Gramella forsetii)
WP_035715687.1	M23B	subfamily M23B non-peptidase homologues (Psychroflexus torquis)
WP_035715739.1	M16B	YMXG peptidase (Croceibacter atlanticus)
WP_035715761.1	M49	dipeptidyl-peptidase IIIB (Gramella forsetii)
WP_035715858.1	M38	family M38 non-peptidase homologues (Kangiella koreensis)
WP_035715973.1	M75	family M75 unassigned peptidases (Spirosoma linguale)
WP_035715996.1	M01	family M1 unassigned peptidases (Gramella forsetii)
WP_035716074.1	S54	family S54 unassigned peptidases (Gramella forsetii)
WP_035716082.1	M14X	family M14 non-peptidase homologues (Gramella forsetii)
WP_035716228.1	S09X	family S9 unassigned peptidases (Flavobacterium johnsoniae)
WP_035716241.1	S33	family S33 unassigned peptidases (Flavobacterium johnsoniae)
WP_035716376.1	M16B	subfamily M16B non-peptidase homologues (Robiginitalea biformata)
WP_035716379.1	S33	family S33 unassigned peptidases (Gramella forsetii)

Continued on next page

Table S1: (continued)

Accession No.	family	Peptidase or homologue
WP_035716450.1	M79	microcystinase MlrA (Robiginitalea biformata)
WP_035716494.1	M38	family M38 non-peptidase homologues (Gramella forsetii)
WP_035716571.1	C40	spr peptidase (Gramella forsetii)
WP_035716572.1	S41A	C-terminal processing peptidase-1 (Gramella forsetii)
WP_035716608.1	S41A	subfamily S41A unassigned peptidases (Gramella forsetii)
WP_035716636.1	M28D	carboxypeptidase Q (Gramella forsetii)
WP_051199619.1	C01A	subfamily C1A unassigned peptidases (Plesiocystis pacifica)
WP_051199635.1	M23B	Mername-AA292 peptidase (Psychroflexus torquis)
WP_051199679.1	S33	family S33 unassigned peptidases (Haliscomenobacter hydrossis)
WP_051199699.1	S11	family S11 unassigned peptidases
WP_051199702.1	S13	family S13 unassigned peptidases (Gramella forsetii)
WP_051199761.1	S09A	prolyl endopeptidase (Myxococcus xanthus) (Chlorobium tepidum)
WP_051199789.1	M56	family M56 unassigned peptidases (Gramella forsetii)
WP_051199795.1	S12	family S12 unassigned peptidases
WP_051199799.1	M38	family M38 non-peptidase homologues (Gramella forsetii)

Table S2: Simple peptidases inhibitors in the genome of *Gramella echinicola* DSM 19838^{T}.

Accession No.	family	Simple peptidase inhibitors
WP_026932781.1	I39	family I39 unassigned peptidase inhibitors (Spirosoma linguale)
WP_026932904.1	I87	family I87 unassigned peptidase inhibitors (Cucumis melo)
WP_026932927.1	I39	family I39 unassigned peptidase inhibitors (Capnocytophaga ochracea)
WP_026933047.1	I39	family I39 unassigned peptidase inhibitor homologues (Pedobacter heparinus)
WP_026933083.1	I39	family I39 unassigned peptidase inhibitor homologues (Pedobacter heparinus)
WP_026933211.1	I39	family I39 unassigned peptidase inhibitors (Spirosoma linguale)
WP_026933535.1	I39	family I39 unassigned peptidase inhibitors (Spirosoma linguale)
WP_026933538.1	I39	family I39 unassigned peptidase inhibitor homologues (Spirosoma linguale)
WP_026933541.1	I39	family I39 unassigned peptidase inhibitors (Chitinophaga pinensis)
WP_026933779.1	I39	family I39 unassigned peptidase inhibitors (Spirosoma linguale)
WP_026934059.1	I39	family I39 unassigned peptidase inhibitors (Capnocytophaga ochracea)
WP_026934120.1	I39	family I39 unassigned peptidase inhibitor homologues (Chitinophaga pinensis)
WP_026934472.1	I39	family I39 unassigned peptidase inhibitors (Spirosoma linguale)
WP_026934518.1	I39	family I39 unassigned peptidase inhibitor homologues (Pedobacter heparinus)
WP_026934636.1	I04	family I4 unassigned peptidase inhibitors (Sulfolobus acidocaldarius)
WP_026934647.1	I39	family I39 unassigned peptidase inhibitors (Spirosoma linguale)

Continued on next page

Table S2: (continued)

Accession No.	family	Simple peptidase inhibitors
WP_026934775.1	I39	family I39 unassigned peptidase inhibitor homologues (Dyadobacter fermentans)
WP_026935065.1	I39	family I39 unassigned peptidase inhibitor homologues (Spirosoma linguale)
WP_026935302.1	I39	family I39 unassigned peptidase inhibitor homologues (Dyadobacter fermentans)
WP_035716629.1	I43	family I43 unassigned peptidase inhibitors (Pedosphaera parvula)
WP_051199809.1	I39	family I39 unassigned peptidase inhibitors (Salinibacter ruber)
WP_026932904.1	I87	family I87 unassigned peptidase inhibitors (Cucumis melo)
WP_026932927.1	I39	family I39 unassigned peptidase inhibitors (Capnocytophaga ochracea)
WP_026933047.1	I39	family I39 unassigned peptidase inhibitor homologues (Pedobacter heparinus)
WP_026933083.1	I39	family I39 unassigned peptidase inhibitor homologues (Pedobacter heparinus)
WP_026933211.1	I39	family I39 unassigned peptidase inhibitors (Spirosoma linguale)
WP_026933535.1	I39	family I39 unassigned peptidase inhibitors (Spirosoma linguale)
WP_026933538.1	I39	family I39 unassigned peptidase inhibitor homologues (Spirosoma linguale)
WP_026933541.1	I39	family I39 unassigned peptidase inhibitors (Chitinophaga pinensis)
WP_026933779.1	I39	family I39 unassigned peptidase inhibitors (Spirosoma linguale)
WP_026934059.1	I39	family I39 unassigned peptidase inhibitors (Capnocytophaga ochracea)
WP_026934120.1	I39	family I39 unassigned peptidase inhibitor homologues (Chitinophaga pinensis)
WP_026934472.1	I39	family I39 unassigned peptidase inhibitors (Spirosoma linguale)
WP_026934518.1	I39	family I39 unassigned peptidase inhibitor homologues (Pedobacter heparinus)
WP_026934636.1	I04	family I4 unassigned peptidase inhibitors (Sulfolobus acidocaldarius)
WP_026934647.1	I39	family I39 unassigned peptidase inhibitors (Spirosoma linguale)
WP_026934775.1	I39	family I39 unassigned peptidase inhibitor homologues (Dyadobacter fermentans)
WP_026935065.1	I39	family I39 unassigned peptidase inhibitor homologues (Spirosoma linguale)
WP_026935302.1	I39	family I39 unassigned peptidase inhibitor homologues (Dyadobacter fermentans)
WP_035716629.1	I43	family I43 unassigned peptidase inhibitors (Pedosphaera parvula)
WP_051199809.1	I39	family I39 unassigned peptidase inhibitors (Salinibacter ruber)

Table S3: Peptidases or homologues in the genome of *Gramella portivictoriae* DSM 23547^{T}.

Accession No.	family	Peptidase or homologue
WP_026913590.1	S12	family S12 unassigned peptidases (Zunongwangia profunda)
WP_026913596.1	U32	family U32 unassigned peptidases (Elizabethkingia anophelis)
WP_026913608.1	M23B	subfamily M23B non-peptidase homologues (Psychroflexus torquis)
WP_026913639.1	M23B	subfamily M23B unassigned peptidases (Gramella forsetii)
WP_026913642.1	M23B	subfamily M23B non-peptidase homologues (Bacillus subtilis)
WP_026913664.1	S41A	CtpC peptidase (Kordia algicida)
WP_026913666.1	M01	family M1 unassigned peptidases (Gramella forsetii)
WP_026913667.1	S08A	subfamily S8A unassigned peptidases (Zunongwangia profunda)
WP_026913674.1	C40	NlpC protein (Escherichia coli) (Cellulophaga lytica)
WP_026913761.1	S09A	prolyl oligopeptidase (Gramella forsetii)
WP_026913778.1	S09X	family S9 unassigned peptidases (Gramella forsetii)
WP_026913781.1	U32	family U32 unassigned peptidases (Chthonomonas calidirosea)
WP_026913784.1	C44	family C44 unassigned peptidases (Gramella forsetii)
WP_026913804.1	T02	At3g16150 (Arabidopsis thaliana) (Gramella forsetii)
WP_026913837.1	S33	family S33 unassigned peptidases (Gramella forsetii)
WP_026913846.1	S09B	subfamily S9B unassigned peptidases (Gramella forsetii)
WP_026913847.1	M28D	subfamily M28D unassigned peptidases (Zunongwangia profunda)
WP_026913874.1	S41A	C-terminal processing peptidase-3 (Zunongwangia profunda)
WP_026913929.1	M23B	Mername-AA292 peptidase (Gramella forsetii)
WP_026913930.1	S16	DNA repair protein RadA (Escherichia coli) (Gramella forsetii)
WP_026913937.1	C44	glucosamine-fructose-6-phosphate aminotransferase
WP_026913940.1	M61	family M61 unassigned peptidases (Gramella forsetii)
WP_026913970.1	M16B	subfamily M16B non-peptidase homologues (Cellulophaga algicola)
WP_026913971.1	M16B	subfamily M16B non-peptidase homologues (Gramella forsetii)
WP_026914029.1	M20F	subfamily M20F unassigned peptidases (Gramella forsetii)
WP_026914044.1	M56	family M56 unassigned peptidases (Gramella forsetii)
WP_026914072.1	M38	family M38 non-peptidase homologues (Gramella forsetii)
WP_026914078.1	S06	family S6 unassigned peptidases (Flavobacterium johnsoniae)
WP_026914098.1	T02	isoaspartyl dipeptidase (threonine type) (Gramella forsetii)
WP_026914101.1	S33	family S33 unassigned peptidases (Ignavibacterium album)
WP_026914104.1	S33	family S33 unassigned peptidases (Pseudomonas mendocina)
WP_026914178.1	S33	family S33 non-peptidase homologues (Gramella forsetii)
WP_026914183.1	S14	peptidase Clp (type 1) (Gramella forsetii)
WP_026914190.1	M23B	Mername-AA292 peptidase (Gramella forsetii)
WP_026914243.1	S24	family S24 unassigned peptidases (Zunongwangia profunda)
WP_026914248.1	M38	family M38 non-peptidase homologues (Gramella forsetii)
WP_026914288.1	S33	family S33 unassigned peptidases (Gramella forsetii)
WP_026914331.1	S09B	subfamily S9B unassigned peptidases (Muricauda ruestringensis)

Continued on next page

Table S3: (continued)

Accession No.	family	Peptidase or homologue
WP_026914335.1	S09X	family S9 non-peptidase homologues (Cellulophaga algicola)
WP_026914373.1	M24A	methionyl aminopeptidase 1 (Escherichia-type) (Gramella forsetii)
WP_026914377.1	M48C	subfamily M48C unassigned peptidases
WP_026914381.1	S14	peptidase Clp (type 5) (Gramella forsetii)
WP_026914396.1	M57	prtB g.p. (Myxococcus xanthus) (Gillisia limnaea)
WP_026914447.1	M23B	subfamily M23B unassigned peptidases (Gillisia limnaea)
WP_026914457.1	C44	family C44 unassigned peptidases (Zunongwangia profunda)
WP_026914522.1	M97	family M97 unassigned peptidases (Gramella forsetii)
WP_026914542.1	S41A	C-terminal processing peptidase-1 (Gramella forsetii)
WP_026914546.1	C40	spr peptidase (Gramella forsetii)
WP_026914551.1	S09B	dipeptidyl-peptidase 4 (bacteria-type 2) (Zunongwangia profunda)
WP_026914690.1	A28	family A28 unassigned peptidases
WP_026914706.1	M13	Zmp1 peptidase (Mycobacterium-type) (Gramella forsetii)
WP_026914719.1	M28X	family M28 unassigned peptidases (Gramella forsetii)
WP_026914737.1	S33	SCO7095-type peptidase (Gramella forsetii)
WP_026914738.1	M24B	subfamily M24B unassigned peptidases (Gramella forsetii)
WP_026914759.1	U69	family U69 unassigned peptidases (Vibrio furnissii)
WP_026914763.1	M23B	Mername-AA292 peptidase
WP_026914772.1	M14X	family M14 non-peptidase homologues (Croceibacter atlanticus)
WP_026914776.1	A08	signal peptidase II (Gramella forsetii)
WP_026914790.1	M50B	subfamily M50B unassigned peptidases (Gramella forsetii)
WP_026914807.1	S33	family S33 unassigned peptidases (Stigmatella aurantiaca)
WP_026914815.1	M16B	At5g56730 (Arabidopsis thaliana)-like peptidase (Lacinutrix sp. 5H-3-7-4)
WP_026914820.1	S26A	subfamily S26A unassigned peptidases (Gramella forsetii)
WP_026914823.1	S54	family S54 unassigned peptidases (Gramella forsetii)
WP_026914824.1	S54	RhoII peptidase (Haloferax volcanii) and similar (Gramella forsetii)
WP_026914838.1	M20C	Pep581 peptidase (Gramella forsetii)
WP_026914840.1	M38	family M38 non-peptidase homologues (Gramella forsetii)
WP_026914841.1	M38	family M38 non-peptidase homologues (Gramella forsetii)
WP_026914843.1	M48A	zmpste24 g.p. (Dictyostelium discoideum-type) (Gramella forsetii)
WP_026914898.1	S01B	At5g27660 (Arabidopsis thaliana) (Gramella forsetii)
WP_026914943.1	M24B	subfamily M24B unassigned peptidases (Bizionia argentinensis)
WP_026914959.1	S24	UmuD protein (Flavobacterium johnsoniae)
WP_026914961.1	M28A	subfamily M28A non-peptidase homologues (Gillisia limnaea)
WP_026914987.1	M15D	vanX D-Ala-D-Ala dipeptidase (Gramella forsetii)
WP_026915005.1	S01C	DegQ peptidase (Gramella forsetii)
WP_026915010.1	S08A	subtilisin NAT
WP_026915014.1	M14B	subfamily M14B non-peptidase homologues (Dokdonia donghaensis)
WP_026915039.1	M23B	subfamily M23B unassigned peptidases (Gramella forsetii)
WP_026915042.1	M23B	subfamily M23B unassigned peptidases (Polaribacter sp. MED152)
WP_026915054.1	S09X	family S9 unassigned peptidases (Gramella forsetii)
WP_026915056.1	M48C	subfamily M48C unassigned peptidases (Gramella forsetii)
WP_026915061.1	S16	Lon peptidase (type 4) (Gramella forsetii)
WP_026915067.1	S09X	family S9 non-peptidase homologues (Gramella forsetii)

Continued on next page

Table S3: (continued)

Accession No.	family	Peptidase or homologue
WP_026915073.1	S41A	subfamily S41A unassigned peptidases (Gramella forsetii)
WP_026915074.1	S09X	family S9 non-peptidase homologues (Spirosoma linguale)
WP_026915090.1	C44	glutamate synthase (alpha chain) precursor (Gramella forsetii)
WP_026915107.1	M28E	subfamily M28E unassigned peptidases (Gramella forsetii)
WP_026915122.1	M03A	subfamily M3A unassigned peptidases (Gramella forsetii)
WP_026915138.1	S54	family S54 unassigned peptidases (Gramella forsetii)
WP_026915170.1	C26	family C26 non-peptidase homologues (Gramella forsetii)
WP_026915199.1	S33	family S33 unassigned peptidases (Gramella forsetii)
WP_026915217.1	S66	family S66 unassigned peptidases (Gramella forsetii)
WP_026915218.1	S13	family S13 unassigned peptidases (Gillisia sp. CBA3202)
WP_026915221.1	C44	AsnB protein (Gramella forsetii)
WP_026915235.1	S51	alpha-aspartyl dipeptidase (eukaryote) (Gramella forsetii)
WP_026915239.1	M01	family M1 unassigned peptidases (Gramella forsetii)
WP_026915271.1	M41	Afg3-like protein 2 (Gramella forsetii)
WP_026915277.1	M14X	family M14 non-peptidase homologues (Polaribacter sp. MED152)
WP_026915326.1	S09C	subfamily S9C unassigned peptidases (Teredinibacter turnerae)
WP_026915348.1	M38	family M38 non-peptidase homologues (Spirosoma linguale)
WP_026915356.1	M14X	family M14 non-peptidase homologues (Gramella forsetii)
WP_026915364.1	M20B	peptidase T (Gramella forsetii)
WP_026915369.1	M75	family M75 unassigned peptidases (Spirosoma linguale)
WP_026915418.1	N11	family N11 unassigned peptide lyases
WP_026915432.1	S33	family S33 non-peptidase homologues (Gramella forsetii)
WP_026915443.1	S09D	glutamyl endopeptidase C (Gramella forsetii)
WP_026915453.1	M01	family M1 unassigned peptidases (Gramella forsetii)
WP_026915458.1	M20D	carboxypeptidase Ss1 (Gramella forsetii)
WP_026915463.1	S33	CPO-A2 (Streptomyces aureofaciens)-type chloroperoxidase (Gramella forsetii)
WP_026915472.1	M79	family M79 unassigned peptidases (Gramella forsetii)
WP_026915475.1	M24X	family M24 non-peptidase homologues (Leeuwenhoekiella blandensis)
WP_026915476.1	M01	family M1 unassigned peptidases (Gramella forsetii)
WP_026915506.1	M14B	subfamily M14B non-peptidase homologues (Dyadobacter fermentans)
WP_026915540.1	M16B	subfamily M16B non-peptidase homologues (Polaribacter sp. MED152)
WP_026915541.1	M14X	family M14 non-peptidase homologues (Gramella forsetii)
WP_026915545.1	C26	GMP synthase (Gramella forsetii)
WP_026915546.1	C40	spr peptidase (Thermodesulfobacterium sp. OPB45)
WP_026915550.1	S08A	subfamily S8A unassigned peptidases (Gramella forsetii)
WP_026915555.1	C26	CTP synthetase (Zunongwangia profunda)
WP_026915565.1	S09C	dpf-6 g.p. (Caenorhabditis elegans) (Gramella forsetii)
WP_026915567.1	S09X	family S9 non-peptidase homologues (Gramella forsetii)
WP_026915573.1	C82	family C82 unassigned peptidases (Chlorobium phaeobacteroides)
WP_026915585.1	S33	family S33 non-peptidase homologues (Gramella forsetii)
WP_026915602.1	S09X	family S9 unassigned peptidases (Gramella forsetii)
WP_026915624.1	S16	family S16 non-peptidase homologues (Zunongwangia profunda)
WP_026915647.1	M20A	BT3549 putative peptidase (Gramella forsetii)
WP_026915684.1	M12A	vertebrate tolloid-like 1 protein (Gramella forsetii)
WP_026915685.1	M12A	blastula protease 10 (Paracentrotus) (Gramella forsetii)
WP_026915689.1	S54	family S54 unassigned peptidases (Gramella forsetii)

Continued on next page

Table S3: (continued)

Accession No.	family	Peptidase or homologue
WP_026915748.1	S10	family S10 unassigned peptidases (Gramella forsetii)
WP_026915750.1	M61	family M61 unassigned peptidases (Gramella forsetii)
WP_026915758.1	T03	gamma-glutamyltransferase 1 (bacterial-type) (Gramella forsetii)
WP_026915761.1	M03A	subfamily M3A unassigned peptidases (Gramella forsetii)
WP_026915768.1	C56	family C56 non-peptidase homologues (Gramella forsetii)
WP_026915813.1	C56	family C56 non-peptidase homologues (Spirosoma linguale)
WP_026915847.1	M42	family M42 unassigned peptidases (Gramella forsetii)
WP_026915869.1	S33	family S33 unassigned peptidases (Gramella forsetii)
WP_026915881.1	S12	family S12 unassigned peptidases (Psychroflexus torquis)
WP_026915913.1	M23B	subfamily M23B non-peptidase homologues (Zunongwangia profunda)
WP_026915983.1	S09X	family S9 unassigned peptidases (Gramella forsetii)
WP_026916002.1	M28E	subfamily M28E non-peptidase homologues (Gramella forsetii)
WP_026916007.1	M43B	ulilysin (Gramella forsetii)
WP_026916013.1	C56	KIAA0361 protein (Homo sapiens-type) (Gramella forsetii)
WP_026916028.1	S33	family S33 unassigned peptidases (Flavobacterium johnsoniae)
WP_026916032.1	C26	dihydro-orotase (N-terminal unit) (Homo sapiens-type) (Ailuropoda melanoleuca)
WP_026916037.1	S12	family S12 unassigned peptidases (Gramella forsetii)
WP_026916085.1	S09X	family S9 unassigned peptidases (Capnocytophaga ochracea)
WP_026916087.1	M38	family M38 non-peptidase homologues (Zunongwangia profunda)
WP_026916116.1	M14X	family M14 non-peptidase homologues (Gramella forsetii)
WP_026916121.1	C56	PfpI peptidase (Gramella forsetii)
WP_026916135.1	C40	family C40 unassigned peptidases (Gramella forsetii)
WP_026916163.1	M19	family M19 unassigned peptidases (Gramella forsetii)
WP_026916194.1	M16B	subfamily M16B non-peptidase homologues (Lacinutrix sp. 5H-3-7-4)
WP_026916234.1	S12	family S12 unassigned peptidases (Gramella forsetii)
WP_026916252.1	C45	family C45 unassigned peptidases (Flavobacterium johnsoniae)
WP_026916279.1	S09X	family S9 non-peptidase homologues (Spirosoma linguale)
WP_026916283.1	S09B	subfamily S9B unassigned peptidases (Haliscomenobacter hydrossis)
WP_026916291.1	C26	imidazole glycerol phosphate synthase subunit HisH (Prochlorococcus marinus)
WP_026916293.1	C26	imidazole glycerol phosphate synthase subunit HisH (Gramella forsetii)

Table S4: Simple peptidases inhibitors in the genome of *Gramella portivictoriae* DSM 23547^{T}.

Accession No.	family	Simple peptidase inhibitors
WP_026913652.1	I87	family I87 unassigned peptidase inhibitors (Thiorhodospira sibirica)
WP_026913697.1	I39	family I39 unassigned peptidase inhibitor homologues (Dyadobacter fermentans)

Continued on next page

Table S4: (continued)

Accession No.	family	Simple peptidase inhibitors
WP_026913703.1	I39	family I39 unassigned peptidase inhibitor homologues (Capnocytophaga ochracea)
WP_026913735.1	I39	family I39 unassigned peptidase inhibitor homologues (Pedobacter heparinus)
WP_026913855.1	I39	family I39 unassigned peptidase inhibitors (Capnocytophaga ochracea)
WP_026913967.1	I39	family I39 unassigned peptidase inhibitors (Chitinophaga pinensis)
WP_026914327.1	I39	family I39 unassigned peptidase inhibitor homologues (Dyadobacter fermentans)
WP_026914517.1	I39	family I39 unassigned peptidase inhibitors (Capnocytophaga ochracea)
WP_026914780.1	I39	family I39 unassigned peptidase inhibitors (Spirosoma linguale)
WP_026914910.1	I39	family I39 unassigned peptidase inhibitors (Spirosoma linguale)
WP_026914937.1	I39	family I39 unassigned peptidase inhibitor homologues (Dyadobacter fermentans)
WP_026915140.1	I39	family I39 unassigned peptidase inhibitors (Chitinophaga pinensis)
WP_026915143.1	I39	family I39 unassigned peptidase inhibitor homologues (Dyadobacter fermentans)
WP_026915301.1	I39	family I39 unassigned peptidase inhibitor homologues (Flavobacterium johnsoniae)
WP_026915614.1	I39	family I39 unassigned peptidase inhibitors (Chitinophaga pinensis)
WP_026915991.1	I39	family I39 unassigned peptidase inhibitors (Spirosoma linguale)
WP_026916005.1	I04	family I4 unassigned peptidase inhibitors (Sulfolobus acidocaldarius)

Table S5: Carbohydrate active enzymes (CAZymes) in the genome of *Gramella echinicola* DSM 19838^{T}. *genes attributed to an enzyme class, but not to a family.

Accession No.	family	Annotation
WP_026932676.1	GT2	two-component system sensor histidine kinase/response regulator (Bacteroides thetaiotaomicron VPI-5482)
WP_026932677.1	CE11	UDP-3-O-(3-hydroxymyristoyl) N-acetylglucosamine deacetylase (Brachyspira pilosicoli 95/1000)
WP_026932743.1	GH84	aminopeptidase P homologue(M24 family) (Schistosoma mansoni)
WP_026932746.1	GH13-8	hypothetical protein Csp3 JD02.017 (Caenorhabditis angaria)
WP_026932826.1	CBM6	Carbohydrate binding family 6 (Emticicia oligotrophica DSM 17448)
WP_026932838.1	GT19	hypothetical protein BRDCF 04625 (Bacteroides sp. CF50)
WP_026932848.1	GT51	bifunctional transglycosylase/transpeptidase penicillin-binding protein (Gramella forsetii KT0803)
WP_026932879.1	GT9	RfaQ-like lipopolysaccharide core biosynthesis glycosyl transferase (Gramella forsetii KT0803)
WP_026932880.1	GT2	conserved hypothetical protein (Azoarcus sp. BH72)
WP_026932884.1	GT4	glycosyl transferases group 1 family protein (Janthinobacterium agaricidamnosum NBRC 102515 = DSM 9628)
WP_026932885.1	GT2	TuaG-like glycosyl transferase (Gramella forsetii KT0803)
WP_026932886.1	GH*	nucleoside diphosphate epimerase (Celeribacter indicus)
WP_026932887.1	GT2	dTDP-4-dehydrorhamnose reductase (Pseudomonas sp. MT-1)
WP_026932891.1	CBM50	metalloendopeptidase-like membrane protein (Desulfotomaculum gibsoniae DSM 7213)
WP_026932894.1	CBM50	membrane-bound lytic murein transglycosylase (Gramella forsetii KT0803)
WP_026932919.1	CBM5	SptC (Natrinema sp. J7-2)
WP_026932926.1	CE9	N-acetylglucosamine-6-phosphate deacetylase/N-acetylmuramic acid 6-phosphate etherase (Acidiphilium multivorum AIU301)
WP_026932929.1	GH*	hypothetical protein AMK43 02675 (Leptotrichia sp. oral taxon 212)
WP_026932932.1	GT2	cell wall-associated hydrolase, invasion-associated protein (Kytococcus sedentarius DSM 20547)
WP_026932937.1	GH89	putative alpha-N-acetylglucosaminidase (Xanthomonas fuscans subsp. fuscans)
WP_026932938.1	GH3	Beta-N-acetylhexosaminidase (Cellulophaga lytica DSM 7489)
WP_026932953.1	GT51	monofunctional biosynthetic peptidoglycan transglycosylase (Gramella forsetii KT0803)
WP_026932955.1	GT4	ABC transporter related (Desulfovibrio desulfuricans subsp. desulfuricans str. ATCC 27774)
WP_026932974.1	CBM6	agarase, partial (Gilvimarinus polysaccharolyticus)
WP_026933024.1	CBM50	LysM domain/BON superfamily protein (Gramella forsetii KT0803)
WP_026933026.1	GH99	mannosidase, endo-alpha, putative (Schistosoma mansoni)
WP_026933035.1	GH97	alpha-glucosidase SusB (Psychroflexus torquis ATCC 700755)
WP_026933050.1	CE8	Pectinesterase (Flavobacteriaceae bacterium 3519-10)
WP_026933065.1	GH28	Glycoside hydrolase precursor, family 28 (Flavobacterium branchiophilum FL-15)

Continued on next page

Table S5: (continued)

Accession No.	family	Annotation
WP_026933066.1	GH105	unsaturated rhamnogalacturonyl hydrolase, putative, urh105A (Cellvibrio japonicus Ueda107)
WP_026933067.1	GH105	Rhamnogalacturonides degradation protein RhiN (Flavobacteriaceae bacterium 3519-10)
WP_026933068.1	GH43	glycoside hydrolase (Pontibacter korlensis)
WP_026933069.1	CE12	GDSL family lipase (Pontibacter korlensis)
WP_026933071.1	GH28	Glycoside hydrolase family 28 (Flavobacterium johnsoniae UW101)
WP_026933072.1	GH35	mannonate dehydratase (Chitinophaga pinensis DSM 2588)
WP_026933085.1	GH16	beta-glucanase (Glaciecola nitratireducens FR1064)
WP_026933086.1	GH16	beta-glucanase precursor (Maribacter sp. HTCC2170)
WP_026933087.1	GH16	Endo-1,4-beta-xylanase A precursor (Altererythrobacter epoxidivorans)
WP_026933088.1	GH3	beta-glucosidase (Gramella forsetii KT0803)
WP_026933089.1	GH16	glycosyl hydrolase, family 16 (Gramella forsetii KT0803)
WP_026933091.1	GH13	alpha amylase catalytic region (Eubacterium limosum KIST612)
WP_026933108.1	PL1-5	pectin methylesterase (Alteromonas australica)
WP_026933110.1	GT2	two-component system sensor histidine kinase/response regulator, hybrid(one component system) (Bacteroides thetaiotaomicron VPI-5482)
WP_026933112.1	GH130	glycosidase related protein (Pseudopedobacter saltans DSM 12145)
WP_026933123.1	AA2	peroxidase (Salmonella enterica subsp. enterica serovar Typhi)
WP_026933134.1	CE14	LmbE family protein (Sphingobacterium sp. PM2-P1-29)
WP_026933135.1	GT2	glycosyl transferase (Chamaesiphon minutus PCC 6605)
WP_026933139.1	GT2	riboflavin biosynthesis protein RibF (Haliscomenobacter hydrossis DSM 1100)
WP_026933141.1	GH95	hypothetical protein AN3106.2 (Aspergillus nidulans FGSC A4)
WP_026933171.1	CBM50	peptidase (Brevibacillus laterosporus)
WP_026933177.1	GT5	glycogen synthase N-terminal domain-like protein (Gramella forsetii KT0803)
WP_026933207.1	GH16	putative A/G-specific adenine glycosylase (Bifidobacterium bifidum ATCC 29521 = JCM 1255 = DSM 20456)
WP_026933233.1	GT2	glycosyl transferase family 2 (Chlorobium limicola DSM 245)
WP_026933236.1	GH15	glycoside hydrolase 15-related (Chlorobaculum parvum NCIB 8327)
WP_026933237.1	GT2	glycosyl transferase, family 2 (Gramella forsetii KT0803)
WP_026933238.1	GH113	conserved hypothetical protein, secreted (Gramella forsetii KT0803)
WP_026933241.1	GT2	RfbJ-like lipopolysaccharide biosynthesis glycosyl transferase (Gramella forsetii KT0803)
WP_026933256.1	GH92	alpha-1,2-mannosidase, putative (Prevotella dentalis DSM 3688)
WP_026933278.1	CE*	Probable esterase precursor (Flavobacterium branchiophilum FL-15)
WP_026933300.1	GT32	glycosyltransferase sugar-binding region containing DXD motif (Beijerinckia indica subsp. indica ATCC 9039)
WP_026933301.1	GH43	fibronectin type III repeat domain containing secreted glycoside hydrolase, family 43 (Gramella forsetii KT0803)
WP_026933307.1	CBM12	dioxygenase (Streptomyces collinus Tu 365)

Continued on next page

Table S5: (continued)

Accession No.	family	Annotation
WP_026933364.1	GT2	dolichol-phosphate mannosyltransferase family protein (Gramella forsetii KT0803)
WP_026933373.1	GT2	two-component system sensor histidine kinase/response regulator, hybrid(one component system) (Bacteroides thetaiotaomicron VPI-5482)
WP_026933374.1	GT*	hypothetical protein CA2559 01800 (Croceibacter atlanticus HTCC2559)
WP_026933375.1	GT30	3-deoxy-D-manno-octulosonic-acid transferase (Fusobacterium nucleatum subsp. polymorphum)
WP_026933403.1	CE4	protein containing polysaccharide deacetylase domain (Gramella forsetii KT0803)
WP_026933408.1	PL4-3	hypothetical protein AN4139.2 (Aspergillus nidulans FGSC A4)
WP_026933443.1	GT4	ABC transporter related (Desulfovibrio desulfuricans subsp. desulfuricans str. ATCC 27774)
WP_026933476.1	CBM50	peptigoglycan-binding protein LysM (Treponema sp. OMZ 838)
WP_026933485.1	CBM50	mannosyl-glycoprotein endo-beta-N-acetylglucosamidase family protein (Gramella forsetii KT0803)
WP_026933486.1	GT2	amino acid adenylation enzyme/thioester reductase family protein (Chamaesiphon minutus PCC 6605)
WP_026933505.1	GT4	Mannose-1-phosphate guanylyltransferase(GDP)(plasmid) (Burkholderia phymatum STM815)
WP_026933507.1	GT4	ABC transporter related (Desulfovibrio desulfuricans subsp. desulfuricans str. ATCC 27774)
WP_026933510.1	GT2	glycosyl transferase family 2 (Siansivirga zeaxanthinifaciens CC-SAMT-1)
WP_026933569.1	GT5	glycogen synthase (Gramella forsetii KT0803)
WP_026933571.1	GH*	putative secreted protein (Chitinophaga pinensis DSM 2588)
WP_026933574.1	CBM16	hypothetical protein PBOR 19105 (Paenibacillus borealis)
WP_026933605.1	GT28	cell division protein FtsW (Kribbella flavida DSM 17836)
WP_026933606.1	GT28	undecaprenyldiphospho-muramoylpentapeptide beta-N-acetylglucosaminyltransferase (Gramella forsetii KT0803)
WP_026933607.1	GT28	UDP-N-acetylmuramate/alanine ligase (Cellulomonas flavigena DSM 20109)
WP_026933618.1	GH3	glycoside hydrolase family 3 domain protein (Sulfurimonas autotrophica DSM 16294)
WP_026933621.1	GT4	asparagine synthase(glutamine-hydrolyzing) (Pseudonocardia dioxanivorans CB1190)
WP_026933629.1	GT2	cysteinyl-tRNA synthetase (Thermobaculum terrenum ATCC BAA-798)
WP_026933660.1	GT30	tetraacyldisaccharide 4'-kinase (Acidaminococcus fermentans DSM 20731)
WP_026933661.1	GT2	two-component system sensor histidine kinase/response regulator, hybrid(one component system) (Bacteroides thetaiotaomicron VPI-5482)
WP_026933665.1	GT2	transmembrane family-2 glycosyl transferase (Gramella forsetii KT0803)
WP_026933672.1	GH18	chitinase I, partial (Pantoea dispersa)
WP_026933684.1	AA12	Putative L-sorbosone dehydrogenase(sndh) protein (Sinorhizobium meliloti 1021)
WP_026933706.1	CE11	DDX20 HUMAN Probable ATP-dependent RNA helicase DDX20(ISS) (Ostreococcus tauri)

Continued on next page

Table S5: (continued)

Accession No.	family	Annotation
WP_026933716.1	CE11	UDP-3-O-(3-hydroxymyristoyl) N-acetylglucosamine deacetylase (Desulfomonile tiedjei DSM 6799)
WP_026933723.1	GT2	glycosyl transferase, family 2 (Zunongwangia profunda SM-A87)
WP_026933727.1	GT4	glycosyltransferase (Zunongwangia profunda SM-A87)
WP_026933733.1	GT4	mannose-1-phosphate guanylyltransferase (Escherichia coli)
WP_026933748.1	CBM32	hypothetical protein R50912 26740 (Paenibacillus sp. FSL R5-0912)
WP_026933765.1	GT66	dolichyl-diphosphooligosaccharide–protein glycotransferase (Candidatus Nitrososphaera gargensis Ga9.2)
WP_026933767.1	GH18	putative bifunctional protein:peroxiredoxin/chitinase ((Clostridium) sordellii)
WP_026933772.1	GT4	ABC transporter related (Desulfovibrio desulfuricans subsp. desulfuricans str. ATCC 27774)
WP_026933808.1	GH3	O-glycosyl hydrolase family protein(homolog to N-acetylglucosaminidase) (Halobacillus halophilus DSM 2266)
WP_026933823.1	CBM13	elongation factor Tu (Streptomyces albulus)
WP_026933895.1	GH2	hypothetical protein AN2824.2 (Aspergillus nidulans FGSC A4)
WP_026933902.1	GT2	WbbL-like lipopolysaccharide biosynthesis glycosyl transferase (Gramella forsetii KT0803)
WP_026933910.1	GT51	peptidoglycan transglycosylase (Halorhodospira halochloris str. A)
WP_026933918.1	GT2	ABC transporter-like protein (Streptomyces sp. 769)
WP_026933926.1	GT2	ABC transporter-like protein (Streptomyces sp. 769)
WP_026933936.1	GT4	putative transferase (Streptomyces griseus subsp. griseus NBRC 13350)
WP_026933937.1	GT2	CDP-glycerol:glycerophosphate glycerophosphotransferase (Cellulophaga lytica)
WP_026933953.1	GH13-11	glycogen debranching protein (Salmonella enterica subsp. enterica serovar Enteritidis str. EC20121179)
WP_026933980.1	CBM50	secreted protein containing LysM domains (Gramella forsetii KT0803)
WP_026934004.1	CE*	conserved hypothetical protein, secreted (Gramella forsetii KT0803)
WP_026934048.1	GH43	aldose 1-epimerase (Prevotella denticola F0289)
WP_026934054.1	GH*	hypothetical protein GFO 2143 (Gramella forsetii KT0803)
WP_026934062.1	GH65	HAD family hydrolase (Anabaena sp. wa102)
WP_026934063.1	GH65	glycoside hydrolase, family 65 (Gramella forsetii KT0803)
WP_026934064.1	GH13	alpha amylase (Gramella forsetii KT0803)
WP_026934065.1	GH13	alpha amylase (Gramella forsetii KT0803)
WP_026934110.1	GH19	Chaperone protein DnaK (Janthinobacterium sp. CG23 2)
WP_026934129.1	CBM13	flavastacin (Corallococcus coralloides DSM 2259)
WP_026934130.1	CBM13	flavastacin (Corallococcus coralloides DSM 2259)
WP_026934162.1	GT2	Glycosyl transferase, group 2 family protein (Flavobacterium branchiophilum FL-15)
WP_026934163.1	GT4	capsular polysaccharide biosynthesis glycosyl transferase (Flammeovirgaceae bacterium 311)
WP_026934164.1	CE4	Predicted xylanase/chitin deacetilase (Clostridium novyi NT)
WP_026934167.1	GT4	glycosyl transferase, group 1 (Gramella forsetii KT0803)
WP_026934170.1	GT2	glycosyl transferase (Echinicola vietnamensis DSM 17526)
WP_026934171.1	GT4	glycosyltransferase (Belliella baltica DSM 15883)

Continued on next page

Table S5: (continued)

Accession No.	family	Annotation
WP_026934172.1	GT2	glycosyl transferase family 2 (Marivirga tractuosa DSM 4126)
WP_026934177.1	GT4	capsular polysaccharide biosynthesis glycosyl transferase (Gramella forsetii KT0803)
WP_026934178.1	GT4	Undecaprenyl-phosphate galactose phosphotransferase (Paenibacillus sp. JDR-2)
WP_026934204.1	GT5	phytochrome b, partial (Mnesithea selloana)
WP_026934211.1	GT4	ABC transporter related (Desulfovibrio desulfuricans subsp. desulfuricans str. ATCC 27774)
WP_026934220.1	GT2	hyaluronan synthase (Gramella forsetii KT0803)
WP_026934237.1	GT28	conserved hypothetical protein (Methylobacterium extorquens CM4)
WP_026934238.1	GT4	hypothetical protein AOC03 12205(plasmid) (Psychrobacter urativorans)
WP_026934239.1	GH17	glycosyltransferase (Azospirillum sp. B510)
WP_026934287.1	GT83	ArnT-like undecaprenyl-phosphate alpha-4-amino-4-deoxy-L-arabinose arabinosyl transferase (Gramella forsetii KT0803)
WP_026934288.1	GH1	mannose-6-phosphate isomerase, class I/beta-galactosidase (Roseburia intestinalis M50/1)
WP_026934314.1	GT2	transmembrane family-2 glycosyl transferase (Gramella forsetii KT0803)
WP_026934317.1	GT2	capsular polysaccharide biosynthesis protein (Sorangium cellulosum So ce56)
WP_026934329.1	GH25	hypothetical protein SALIVB 1060 (Streptococcus salivarius CCHSS3)
WP_026934334.1	GH3	glycoside hydrolase (Nonlabens dokdonensis DSW-6)
WP_026934335.1	GT4	glycosyl transferase, group 1 (Gramella forsetii KT0803)
WP_026934341.1	GT49	predicted protein (Phaeodactylum tricornutum CCAP 1055/1)
WP_026934348.1	GH31	hypothetical protein AN8217.2 (Aspergillus nidulans FGSC A4)
WP_026934403.1	GT4	glycosyl transferase, group 1 (Gramella forsetii KT0803)
WP_026934416.1	GT51	bifunctional transglycosylase/transpeptidase penicillin-binding protein (Zunongwangia profunda SM-A87)
WP_026934430.1	GT2	UDP-glucose 4-epimerase(plasmid) (Cyanothece sp. PCC 7822)
WP_026934444.1	GT2	cell wall-associated hydrolase, invasion-associated protein (Kytococcus sedentarius DSM 20547)
WP_026934446.1	GT51	penicillin-binding protein, partial (uncultured Parcubacteria bacterium Rifle 16ft 4 minimus 37658)
WP_026934471.1	GH72	Rab1 family GTPase(PiYpt1) putative (Albugo laibachii Nc14)
WP_026934473.1	GH13-16	trehalose synthase (Robiginitalea biformata HTCC2501)
WP_026934488.1	CBM*	hypothetical protein GFO 1460 (Gramella forsetii KT0803)
WP_026934489.1	GT4	ABC transporter related (Desulfovibrio desulfuricans subsp. desulfuricans str. ATCC 27774)
WP_026934566.1	AA1	copper oxidase (Mycobacterium abscessus UC22)
WP_026934583.1	GT4	nicotinate-nucleotide pyrophosphorylase(carboxylating) (Flexibacter litoralis DSM 6794)
WP_026934586.1	CE11	DDX20 HUMAN Probable ATP-dependent RNA helicase DDX20(ISS) (Ostreococcus tauri)
WP_026934617.1	GT2	undecaprenyl-phosphate 4-deoxy-4-formamido-L-arabinose transferase (Gramella forsetii KT0803)

Continued on next page

Table S5: (continued)

Accession No.	family	Annotation
WP_026934636.1	GH15	glycosyl hydrolase, family 15 (Gramella forsetii KT0803)
WP_026934637.1	GT20	trehalose 6-phosphate synthase/phosphatase (Gramella forsetii KT0803)
WP_026934668.1	GT66	dolichyl-diphosphooligosaccharide–protein glycotransferase (Candidatus Nitrososphaera gargensis Ga9.2)
WP_026934685.1	GT4	thioredoxin reductase (Inonotus obliquus)
WP_026934744.1	CBM50	trifunctional nucleotide phosphoesterase protein YfkN (Sinorhizobium fredii USDA 257)
WP_026934754.1	GT2	membrane glycosyl transferase, family 2 (Gramella forsetii KT0803)
WP_026934771.1	GH29	Alpha-L-fucosidase, family GH29 / Acetylesterase (Zobellia galactanivorans)
WP_026934774.1	GH32	glycosyl hydrolase, family 32 (Gramella forsetii KT0803)
WP_026934783.1	GT2	predicted sensory transduction regulatory protein (Methanothermobacter marburgensis str. Marburg)
WP_026934802.1	GT51	Multimodular transpeptidase-transglycosylase (Castellaniella defragrans 65Phen)
WP_026934815.1	CE14	conserved hypothetical protein (Gramella forsetii KT0803)
WP_026934827.1	GT47	hypothetical protein VITISV 016911 (Vitis vinifera)
WP_026934833.1	CE*	amidohydrolase (Caulobacter segnis ATCC 21756)
WP_026934836.1	GT66	peptidyl-prolyl cis-trans isomerase(rotamase) - cyclophilin family (Candidatus Nitrososphaera evergladensis SR1)
WP_026934864.1	CBM13	hypothetical protein MFUL124B02 36260 (Myxococcus fulvus 124B02)
WP_026934869.1	GT2	transcriptional regulator (Cellulophaga lytica)
WP_026934873.1	CBM50	5'-Nucleotidase domain protein (Paenibacillus sp. JDR-2)
WP_026934879.1	GT2	sulfatase (Leadbetterella byssophila DSM 17132)
WP_026934884.1	GT2	transmembrane family-2 glycosyl transferase-possibly involved in biofilm formation (Gramella forsetii KT0803)
WP_026934917.1	GH16	glycosyl hydrolase, family 16 (Gramella forsetii KT0803)
WP_026934930.1	CE14	hypothetical protein GFO 1816 (Gramella forsetii KT0803)
WP_026934954.1	GT4	glycosyl transferase, group 1 (Gramella forsetii KT0803)
WP_026934967.1	CE11	LpxC/FabZ bifunctional enzyme (Gramella forsetii KT0803)
WP_026934993.1	GH43	Dipeptidyl aminopeptidase/acylaminoacyl-peptidase (Xanthomonas citri subsp. citri UI6)
WP_026935002.1	GT2	undecaprenyl-phosphate 4-deoxy-4-formamido-L-arabinose transferase-like protein (Gramella forsetii KT0803)
WP_026935031.1	GH*	AraC family transcriptional regulator (Clostridium sporogenes)
WP_026935038.1	GH3	glycoside hydrolase family 3 domain protein (Leadbetterella byssophila DSM 17132)
WP_026935046.1	CE1	hypothetical protein plpp0079(plasmid) (Legionella pneumophila str. Paris)
WP_026935058.1	GH2	glycoside hydrolase, family 2 (Gramella forsetii KT0803)
WP_026935060.1	CE*	secreted alpha/beta fold hydrolase-possibly a phospholipase/carboxylesterase (Gramella forsetii KT0803)
WP_026935067.1	GH3	unnamed protein product (Aspergillus oryzae RIB40)
WP_026935085.1	GT2	ABC transporter-like protein (Streptomyces sp. 769)
WP_026935105.1	GT9	glycosyl transferase, family 9 (Burkholderia glumae PG1)
WP_026935134.1	CBM48	hypothetical protein GFO 0282 (Gramella forsetii KT0803)
WP_026935142.1	GT30	3-deoxy-D-manno-octulosonic-acid transferase (Gramella forsetii KT0803)

Continued on next page

Table S5: (continued)

Accession No.	family	Annotation
WP_026935173.1	GT2	ABC transporter-like protein (Streptomyces sp. 769)
WP_026935187.1	GT2	glycosyl transferase (Zunongwangia profunda SM-A87)
WP_026935188.1	GT2	putative intercellular adhesion protein A (Azorhizobium caulinodans ORS 571)
WP_026935225.1	GT2	response regulator receiver modulated diguanylate cyclase (Geodermatophilus obscurus DSM 43160)
WP_026935241.1	GH31	Alpha-glucosidase, family 31 of glycosyl hydrolase (Maribacter sp. HTCC2170)
WP_026935242.1	GH13-9	1,4-alpha-glucan branching enzyme (Gramella forsetii KT0803)
WP_026935243.1	GH13-16	trehalose synthase (Flammeovirgaceae bacterium 311)
WP_026935244.1	GH13-3	alpha-amylase (Winogradskyella sp. PG-2)
WP_026935268.1	GT19	lipid-A-disaccharide synthase (Gramella forsetii KT0803)
WP_026935272.1	GT28	cell division protein FtsW (Kribbella flavida DSM 17836)
WP_026935308.1	CBM13	hypothetical protein N008 02650 (Hymenobacter sp. APR13)
WP_026935362.1	CBM48	glycoside hydrolase family 13 domain protein (Desulfatibacillum alkenivorans AK-01)
WP_026935392.1	GT4	putative glycosyltransferase (Desulfovibrio magneticus RS-1)
WP_026935398.1	GH108	hypothetical protein Z042 16435 (Serratia fonticola RB-25)
WP_026935415.1	CBM13	alpha-L-arabinofuranosidase (Corallococcus coralloides DSM 2259)
WP_026935430.1	GT8	Glycosyl transferase, family 8-glycogenin (Xanthophyllomyces dendrorhous)
WP_026935460.1	CE11	UDP-3-O-(3-hydroxymyristoyl) N-acetylglucosamine deacetylase (Desulfomonile tiedjei DSM 6799)
WP_026935484.1	GT4	ABC transporter related (Desulfovibrio desulfuricans subsp. desulfuricans str. ATCC 27774)
WP_026935487.1	CBM50	teichoic acid ABC transporter ATP-binding protein (Lactococcus lactis subsp. lactis)
WP_026935497.1	GT2	colanic acid biosynthesis glycosyl transferase (Gramella forsetii KT0803)
WP_026935501.1	GT2	glycosyl transferase family 2 (Melioribacter roseus P3M-2)
WP_026935503.1	GT10	glycosyl transferase (Gramella forsetii KT0803)
WP_026935505.1	GT*	glycosyl transferase, family 2 (Gramella forsetii KT0803)
WP_026935509.1	GT*	conserved hypothetical protein, cytidylyltransferase family (uncultured archaeon)
WP_026935510.1	GT4	asparagine synthase(glutamine-hydrolyzing) (Pseudonocardia dioxanivorans CB1190)
WP_026935512.1	GT4	glycosyl transferase, group 1 (Gramella forsetii KT0803)
WP_026935513.1	GT2	glycosyl transferase, family 2 (Gramella forsetii KT0803)
WP_026935514.1	GT2	glycosyl transferase, family 2 (Gramella forsetii KT0803)
WP_026935515.1	GT64	hypothetical protein (Zea mays)
WP_026935517.1	GT4	glycosyl transferase, group 1 (Gramella forsetii KT0803)
WP_026935520.1	GT4	glycosyl transferases group 1 (Gramella forsetii KT0803)
WP_026935521.1	GT4	glycosyl transferases group 1 (Gramella forsetii KT0803)
WP_026935522.1	GT2	putative glycosyltransferase (Moritella viscosa)
WP_026935543.1	GH28	hypothetical protein VITISV 002009 (Vitis vinifera)
WP_035715612.1	CBM6	Carbohydrate binding family 6 (Niastella koreensis GR20-10)
WP_035715653.1	GT2	hypothetical protein SYNPCCN 0346 (Synechocystis sp. PCC 6803 substr. PCC-N)
WP_035715744.1	GT2	transmembrane family-2 glycosyl transferase-possibly involved in biofilm formation (Gramella forsetii KT0803)

Continued on next page

Table S5: (continued)

Accession No.	family	Annotation
WP_035715765.1	GT2	transmembrane family-2 glycosyl transferase-possibly involved in biofilm formation (Gramella forsetii KT0803)
WP_035715773.1	CBM*	hypothetical protein GFO 0138 (Gramella forsetii KT0803)
WP_035715963.1	GH5-42	glycosidase (Zunongwangia profunda SM-A87)
WP_035716021.1	GH13	alpha-amlyase (Cellulophaga baltica 18)
WP_035716058.1	GH23	putative soluble lytic transglycosylase fused to an ABC-type amino acid-binding protein (Owenweeksia hongkongensis DSM 17368)
WP_035716203.1	CE4	membrane protein containing polysaccharide deacetylase domain (Gramella forsetii KT0803)
WP_035716456.1	CBM20	helix-turn-helix domain-containing protein AraC type (Emticicia oligotrophica DSM 17448)
WP_035716516.1	GH3	Beta-glucosidase (Cellulophaga algicola DSM 14237)
WP_035716571.1	CBM50	Putative peptidoglycan binding domain protein (Flavobacterium psychrophilum)
WP_035716629.1	GH8, CBM9	candidate b-glycosidase, CBM9 module, Glycoside Hydrolase Family 8 protein (Cytophaga hutchinsonii ATCC 33406)
WP_035716644.1	CBM50	Teichoic acid ABC transporter (Lactococcus lactis subsp. lactis NCDO 2118)
WP_051199619.1	CBM13	secreted protein (Achlya hypogyna)
WP_051199624.1	CBM57	Fibronectin type III domain protein (Cyclobacterium marinum DSM 745)
WP_051199635.1	CBM50	peptidase M23 (Pseudomonas syringae pv. actinidiae ICMP 18884)
WP_051199650.1	GH20	hypothetical protein WQ53 10285 (Pseudoxanthomonas suwonensis)
WP_051199652.1	GH3	O-glycosyl hydrolase family protein(homolog to N-acetylglucosaminidase) (Halobacillus halophilus DSM 2266)
WP_051199662.1	PL9-1	hypothetical Protein YC6258 05515 (Gynuella sunshinyii YC6258)
WP_051199664.1	PL9-1	hypothetical Protein YC6258 05515 (Gynuella sunshinyii YC6258)
WP_051199666.1	GH*	hypothetical protein Deipr 2087(plasmid) (Deinococcus proteolyticus MRP)
WP_051199683.1	CBM57	putative secreted protein (Maribacter sp. HTCC2170)
WP_051199688.1	CE8	pectin esterase (Pontibacter korlensis)
WP_051199697.1	GH26	putative glycosyltransferase(GT2) (Formosa agariphila KMM 3901)
WP_051199730.1	GT4	glycosyl transferase, group 1 (Gramella forsetii KT0803)
WP_051199731.1	GT9	LPS heptosyltransferase II (Winogradskyella sp. PG-2)
WP_051199735.1	GH74	hypothetical protein VC82 851 (Muricauda lutaonensis)
WP_051199736.1	GH74	hypothetical protein VC82 851 (Muricauda lutaonensis)
WP_051199737.1	GH74	hypothetical protein VC82 851 (Muricauda lutaonensis)
WP_051199738.1	GT10	alpha-1,3-fucosyltransferase (Coraliomargarita akajimensis DSM 45221)
WP_051199739.1	GT2	putative glycosyl transferase family 2 protein (Pyrococcus sp. ST04)
WP_051199744.1	GT2	glycosyl transferase family 2 (Methylobacterium extorquens PA1)
WP_051199780.1	GH37	trehalase (Gramella forsetii KT0803)
WP_051199782.1	GT2	two-component system sensor histidine kinase/response regulator, hybrid(one component system) (Bacteroides thetaiotaomicron VPI-5482)

Continued on next page

Table S5: (continued)

Accession No.	family	Annotation
WP_051199787.1	GT8	Glycosyl transferase, family 8-glycogenin (Xanthophyllomyces dendrorhous)
WP_051199824.1	GT2	glycosyl transferase family 2 (Nonlabens dokdonensis DSW-6)
WP_051199825.1	GT*	glycosyl transferase family 2 (Methylotenera mobilis JLW8)
WP_051199826.1	GT4	glycosyl transferase, family 2 (Trichodesmium erythraeum IMS101)
WP_051199827.1	GT4	exopolysaccharide biosynthesis glycosyltransferase EpsF (Nonlabens marinus S1-08)
WP_051199828.1	GT4	glycosyl transferase, group 1 (Gramella forsetii KT0803)

Table S6: Carbohydrate active enzymes (CAZymes) in the genome of *Gramella portivictoriae* DSM 23547^{T}.

Accession No.	family	Annotation
WP_026913576.1	GH*	glycosyl hydrolase BNR repeat-containing protein (Spirosoma linguale DSM 74)
WP_026913589.1	GT19	hypothetical protein BRDCF_04625 (Bacteroides sp. CF50)
WP_026913593.1	GT51	penicillin-binding protein 1A (Croceibacter atlanticus HTCC2559)
WP_026913628.1	GT2	glycosyl transferase family 2 (Burkholderia multivorans ATCC 17616)
WP_026913632.1	GT4	glycosyl transferases group 1 family protein (Janthinobacterium agaricidamnosum NBRC 102515, DSM 9628)
WP_026913633.1	GT2	TuaG-like glycosyl transferase (Gramella forsetii KT0803)
WP_026913635.1	GT2	dTDP-4-dehydrorhamnose reductase (Pseudomonas stutzeri DSM 10701)
WP_026913639.1	CBM50	peptigoglycan-binding protein LysM (Desulfitobacterium metallireducens DSM 15288)
WP_026913642.1	GH23, CBM50	membrane-bound lytic murein transglycosylase (Gramella forsetii KT0803)
WP_026913667.1	CBM5	SptC (Natrinema sp. J7-2)
WP_026913672.1	CE9	N-acetylglucosamine-6-phosphate deacetylase/N-acetylmuramic acid 6-phosphate etherase (Acidiphilium multivorum AIU301)
WP_026913674.1	CBM50	NLP/P60 (Moorella thermoacetica ATCC 39073)
WP_026913687.1	GT2	transmembrane family-2 glycosyl transferase-possibly involved in biofilm formation (Gramella forsetii KT0803)
WP_026913689.1	GT2	Dolichol-phosphate mannose synthase, family GT2 (Zobellia galactanivorans)
WP_026913691.1	GT2	two-component system sensor histidine kinase/response regulator, hybrid (one component system) (Bacteroides thetaiotaomicron VPI-5482)
WP_026913696.1	GH32	sucrose-6-phosphate hydrolase (Halalkalicoccus jeotgali B3)
WP_026913701.1	GT2	response regulator receiver modulated diguanylate cyclase (Geodermatophilus obscurus DSM 43160)
WP_026913702.1	GH32	glycosyl hydrolase, family 32 (Gramella forsetii KT0803)
WP_026913705.1	GH32, CBM38	putative levanase (Streptomyces scabiei 87.22)
WP_026913715.1	GT2	response regulator receiver modulated diguanylate cyclase (Geodermatophilus obscurus DSM 43160)
WP_026913731.1	PL6-subfamily_1	secreted alginate lyase-like protein (Gramella forsetii KT0803)
WP_026913732.1	PL12	Heparinase II/III family protein (Cellulophaga algicola DSM 14237)
WP_026913733.1	PL17-subfamily_2	heparinase II/III-like protein (Alteromonas sp. SN2)
WP_026913737.1	PL7	secreted alginate lyase-like protein (Gramella forsetii KT0803)
WP_026913738.1	PL7	secreted alginate lyase-like protein (Gramella forsetii KT0803)
WP_026913741.1	GT51	penicillin-binding , 1A family protein (Mesorhizobium huakuii 7653R)
WP_026913757.1	GT51	monofunctional biosynthetic peptidoglycan transglycosylase (Gramella forsetii KT0803)

Continued on next page

Table S6: (continued)

Accession No.	family	Annotation
WP_026913759.1	GT4	ABC transporter related (Desulfovibrio desulfuricans subsp. desulfuricans str. ATCC 27774)
WP_026913780.1	GH86, CBM6	agarase, partial (Gilvimarinus sp. YN3)
WP_026913801.1	GT35	multidrug transporter (Salmonella enterica subsp. enterica serovar Enteritidis str. EC20121177)
WP_026913833.1	CBM50	LysM domain/BON superfamily protein (Gramella forsetii KT0803)
WP_026913835.1	GH99	mannosidase, endo-alpha, putative (Schistosoma mansoni)
WP_026913843.1	GH97	Candidate alpha-glucosidase; Glycoside hydrolase family 97 (Flavobacterium johnsoniae UW101)
WP_026913857.1	GH16	glycoside hydrolase family 16 (Emticicia oligotrophica DSM 17448)
WP_026913858.1	GH16	glycosyl hydrolase, family 16 (Gramella forsetii KT0803)
WP_026913859.1	GH3	beta-glucosidase (Gramella forsetii KT0803)
WP_026913860.1	GH16	glycosyl hydrolase, family 16 (Gramella forsetii KT0803)
WP_026913861.1	GH13	alpha amylase catalytic region (Eubacterium limosum KIST612)
WP_026913888.1	GH130	glycosidase related protein (Pedobacter saltans DSM 12145)
WP_026913891.1	AA0	bifunctional heme catalase-peroxidase (Zunongwangia profunda SM-A87)
WP_026913900.1	GT2	riboflavin biosynthesis protein RibF (Haliscomenobacter hydrossis DSM 1100)
WP_026913902.1	GH95, GH1	hypothetical protein AN3106.2 (Aspergillus nidulans FGSC A4)
WP_026913929.1	CBM50	L-Ala–D-Glu endopeptidase precursor (Brevibacillus laterosporus LMG 15441)
WP_026913935.1	GT5	glycogen synthase N-terminal domain-like protein (Gramella forsetii KT0803)
WP_026913989.1	GT2	glycosyl transferase family 2 (Chlorobium limicola DSM 245)
WP_026913992.1	GH15	glycoside hydrolase 15-related (Chlorobium phaeobacteroides BS1)
WP_026913993.1	GT2	glycosyl transferase, family 2 (Gramella forsetii KT0803)
WP_026913994.1	GH113	conserved hypothetical protein, secreted (Gramella forsetii KT0803)
WP_026913997.1	GT2	RfbJ-like lipopolysaccharide biosynthesis glycosyl transferase (Gramella forsetii KT0803)
WP_026913998.1	GT2	transmembrane family-2 glycosyl transferase-possibly involved in biofilm formation (Gramella forsetii KT0803)
WP_026914018.1	GH32	glycosyl hydrolase, family 32 (Gramella forsetii KT0803)
WP_026914021.1	GT2	hyaluronan synthase (Gramella forsetii KT0803)
WP_026914040.1	GT2	transmembrane family-2 glycosyl transferase (Zunongwangia profunda SM-A87)
WP_026914071.1	GT2	dolichol-phosphate mannosyltransferase family protein (Gramella forsetii KT0803)
WP_026914076.1	PL6	alginate lyase precursor (Formosa agariphila KMM 3901)
WP_026914080.1	GT2	two-component system sensor histidine kinase/response regulator, hybrid (one component system) (Bacteroides thetaiotaomicron VPI-5482)
WP_026914081.1	GT*	putative glycosyl transferase (Aequorivita sublithincola DSM 14238)
WP_026914082.1	GT30	bifunctional glycosyltransferase/methyltransferase (Fusobacterium nucleatum subsp. vincentii 3_1_36A2)

Continued on next page

Table S6: (continued)

Accession No.	family	Annotation
WP_026914108.1	CE4	protein containing polysaccharide deacetylase domain (Gramella forsetii KT0803)
WP_026914118.1	PL4-subfamily_3	hypothetical protein AN4139.2 (Aspergillus nidulans FGSC A4)
WP_026914157.1	GT4	ABC transporter related (Desulfovibrio desulfuricans subsp. desulfuricans str. ATCC 27774)
WP_026914190.1	CBM50	Peptidoglycan-specific endopeptidase, M23 family protein (Borrelia parkeri SLO)
WP_026914198.1	GH73, CBM50	mannosyl-glycoprotein endo-beta-N-acetylglucosamidase family protein (Gramella forsetii KT0803)
WP_026914199.1	GT2	amino acid adenylation enzyme/thioester reductase family protein (Chamaesiphon minutus PCC 6605)
WP_026914246.1	GT30	3-deoxy-D-manno-octulosonic-acid transferase (Gramella forsetii KT0803)
WP_026914250.1	GT2	amino acid adenylation enzyme/thioester reductase family protein (Chamaesiphon minutus PCC 6605)
WP_026914256.1	CBM48	hypothetical protein GFO_0282 (Gramella forsetii KT0803)
WP_026914325.1	GH3	unnamed protein product (Aspergillus oryzae RIB40)
WP_026914331.1	CE*	secreted alpha/beta fold hydrolase-possibly a phospholipase/carboxylesterase (Gramella forsetii KT0803)
WP_026914332.1	GH43	fibronectin type III repeat domain containing secreted glycoside hydrolase, family 43 (Gramella forsetii KT0803)
WP_026914333.1	CE15, GH78	hypothetical protein (uncultured bacterium pUR16A2)
WP_026914334.1	GH2, CBM57	glycoside hydrolase, family 2 (Gramella forsetii KT0803)
WP_026914356.1	CBM57	putative secreted protein (Maribacter sp. HTCC2170)
WP_026914357.1	GH8, CBM9	CHU large protein, candidate b-glycosidase, CBM9 module, Glycoside Hydrolase Family 8 protein (Cytophaga hutchinsonii ATCC 33406)
WP_026914371.1	GT4	putative glycosyltransferase (Desulfovibrio magneticus RS-1)
WP_026914378.1	GH108	hypothetical protein Z042_16435 (Serratia fonticola RB-25)
WP_026914396.1	CBM13	alpha-L-arabinofuranosidase (Corallococcus coralloides DSM 2259)
WP_026914436.1	CE11	UDP-3-O-(3-hydroxymyristoyl) N-acetylglucosamine deacetylase (Desulfomonile tiedjei DSM 6799)
WP_026914451.1	GT4	ABC transporter related (Desulfovibrio desulfuricans subsp. desulfuricans str. ATCC 27774)
WP_026914454.1	CBM50	Teichoic acid ABC transporter (Lactococcus lactis subsp. lactis NCDO 2118)
WP_026914458.1	GT82	bifunctional beta-1,4-N-acetylgalactosaminyltransferase/CMP-Neu5Ac synthase (Campylobacter jejuni subsp. jejuni 00-2426)
WP_026914460.1	GT4	glycosyltransferase (Nonlabens marinus S1-08)
WP_026914463.1	GT2	glycosyltransferase (Zunongwangia profunda SM-A87)
WP_026914466.1	GT2	DegT/DnrJ/EryC1/StrS aminotransferase (Pectobacterium carotovorum subsp. carotovorum PC1)
WP_026914469.1	GT4	glycosyl transferase group 1 (Methanohalobium evestigatum Z-7303)
WP_026914471.1	GT8	animal glycogenin, Glycosyltransferase Family 8-like protein (Ramlibacter tataouinensis TTB310)
WP_026914472.1	GT2	glycosyl transferase (Croceibacter atlanticus HTCC2559)

Continued on next page

Table S6: (continued)

Accession No.	family	Annotation
WP_026914473.1	GT4	hypothetical protein IA05_06040 (Flavobacterium psychrophilum)
WP_026914476.1	GT2	glycosyl transferase, family 2 (Gramella forsetii KT0803)
WP_026914477.1	GT2	glycosyltransferase (Zunongwangia profunda SM-A87)
WP_026914480.1	GT4	glycosyl transferase, group 1 (Gramella forsetii KT0803)
WP_026914481.1	GT4	glycosyl transferase, group 1 (Gramella forsetii KT0803)
WP_026914483.1	GT*	glycosyl transferase, family 2 (Gramella forsetii KT0803)
WP_026914486.1	GT2	glycosyl transferase, family 2 (Gramella forsetii KT0803)
WP_026914489.1	GT4	glycosyl transferase, group 1 (Gramella forsetii KT0803)
WP_026914490.1	GT4	glycosyl transferase, group 1 (Gramella forsetii KT0803)
WP_026914492.1	GT4	glycosyl transferases group 1 (Gramella forsetii KT0803)
WP_026914493.1	GT4	glycosyl transferases group 1 (Gramella forsetii KT0803)
WP_026914494.1	GT2	hypothetical intercellular adhesion protein A (Photobacterium profundum SS9)
WP_026914514.1	GH28	hypothetical protein VITISV_002009 (Vitis vinifera)
WP_026914524.1	AA1	Multicopper oxidase (Elizabethkingia anophelis NUHP1)
WP_026914541.1	GT28	cell division protein FtsW (Kribbella flavida DSM 17836)
WP_026914545.1	GT19	lipid-A-disaccharide synthase (Gramella forsetii KT0803)
WP_026914546.1	CBM50	NLP/P60 protein (Flavobacterium johnsoniae UW101)
WP_026914569.1	GH13-9, CBM48	1,4-alpha-glucan branching enzyme (Gramella forsetii KT0803)
WP_026914570.1	GH31	alpha-glucosidase (Gramella forsetii KT0803)
WP_026914602.1	CBM50	cell wall hydrolase/autolysin (Marinomonas posidonica IVIA-Po-181)
WP_026914671.1	GT2	two-component system sensor histidine kinase/response regulator, hybrid (one component system) (Bacteroides thetaiotaomicron VPI-5482)
WP_026914672.1	CE11	response regulator receiver protein (Brachyspira murdochii DSM 12563)
WP_026914738.1	GH84	aminopeptidase P homologue (M24 family) (Schistosoma mansoni)
WP_026914739.1	GH*	hypothetical protein Celal_1602 (Cellulophaga algicola DSM 14237)
WP_026914742.1	GH13-8, CBM48	hypothetical protein Csp3_JD02.017 (Caenorhabditis angaria)
WP_026914759.1	CE3	hypothetical protein Riv7116_6589 (Rivularia sp. PCC 7116)
WP_026914763.1	CBM50	peptidase M23B (Shewanella pealeana ATCC 700345)
WP_026914808.1	CBM9	CHU large protein (Cytophaga hutchinsonii ATCC 33406)
WP_026914815.1	GH12	insulinase family protein (Mesorhizobium huakuii 7653R)
WP_026914837.1	GT66	peptidyl-prolyl cis-trans isomerase (rotamase) - cyclophilin family (Candidatus Nitrososphaera evergladensis SR1)
WP_026914846.1	GT47	hypothetical protein VITISV_016911 (Vitis vinifera)
WP_026914858.1	CE14	conserved hypothetical protein (Gramella forsetii KT0803)
WP_026914867.1	GT51	Multimodular transpeptidase-transglycosylase (Castellaniella defragrans 65Phen)
WP_026914879.1	GH13, CBM48	maltooligosyl trehalose hydrolase (Cellulophaga algicola DSM 14237)
WP_026914902.1	GH1	beta-galactosidase (Roseburia hominis A2-183)
WP_026914904.1	GH9	glycoside hydrolase family 9 (Opitutus terrae PB90-1)
WP_026914906.1	GH27	alpha-galactosidase (Melioribacter roseus P3M-2)
WP_026914911.1	GH5-subfamily_2	Endo-beta-1,4-glucanase, family GH5 (Zobellia galactanivorans)
WP_026914912.1	GH39	transcriptional regulator, AraC family (Alkaliphilus oremlandii OhILAs)

Continued on next page

Table S6: (continued)

Accession No.	family	Annotation
WP_026914913.1	GH26	Mannan endo-1,4-beta-mannosidase (Cellulophaga algicola DSM 14237)
WP_026914915.1	GH130	glycosidase, PH1107-related protein (Zunongwangia profunda SM-A87)
WP_026914916.1	GH130	hypothetical protein CCDG5_2018 ((Clostridium) cellulosi)
WP_026914917.1	GH26	Candidate beta-mannanase; Glycoside hydrolase family 26 (Flavobacterium johnsoniae UW101)
WP_026914918.1	GH5-7	Candidate beta-glycosidase; Glycoside hydrolase family 5 (Flavobacterium johnsoniae UW101)
WP_026914919.1	GH3	Candidate beta-glucosidase; Glycoside hydrolase family 3 (Flavobacterium johnsoniae UW101)
WP_026914920.1	CBM35	protein of unknown function (DUF303) (Thermobacillus composti KWC4)
WP_026914939.1	GH63	periplasmic trehalase-like protein (Gramella forsetii KT0803)
WP_026914940.1	GH2	beta-galactosidase (Gramella forsetii KT0803)
WP_026914942.1	GH39	beta-xylosidase XynB (Clostridium saccharoperbutylacetonicum N1-4(HMT))
WP_026914949.1	CE11	UDP-3-O-(3-hydroxymyristoyl) N-acetylglucosamine deacetylase (Desulfomonile tiedjei DSM 6799)
WP_026914971.1	GH16	beta-porphyranase B (GH16) (Formosa agariphila KMM 3901)
WP_026914976.1	GT2	membrane glycosyl transferase, family 2 (Gramella forsetii KT0803)
WP_026914986.1	CBM50	5'-nucleotidase (Paenibacillus mucilaginosus K02)
WP_026915010.1	CBM50	peptidase S8 and S53 subtilisin kexin sedolisin (Halothermothrix orenii H 168)
WP_026915074.1	GH3	OmpA/MotB domain protein (Pedobacter saltans DSM 12145)
WP_026915112.1	GT4	ABC transporter related (Desulfovibrio desulfuricans subsp. desulfuricans str. ATCC 27774)
WP_026915115.1	GT2	glycosyl transferase (Aequorivita sublithincola DSM 14238)
WP_026915131.1	GT28	UDP-N-acetylmuramate/alanine ligase (Cellulomonas flavigena DSM 20109)
WP_026915207.1	GT28	cell division protein FtsW (Kribbella flavida DSM 17836)
WP_026915208.1	GT28	undecaprenyldiphospho-muramoylpentapeptide beta-N-acetylglucosaminyltransferase (Gramella forsetii KT0803)
WP_026915209.1	GT28	UDP-N-acetylmuramate/alanine ligase ((Cellvibrio) gilvus ATCC 13127)
WP_026915228.1	GT2	cysteinyl-tRNA synthetase (Thermobaculum terrenum ATCC BAA-798)
WP_026915255.1	GT30	tetraacyldisaccharide 4'-kinase (Acidaminococcus intestini RyC-MR95)
WP_026915256.1	GT2	two-component system sensor histidine kinase/response regulator, hybrid (one component system) (Bacteroides thetaiotaomicron VPI-5482)
WP_026915260.1	GT2	transmembrane family-2 glycosyl transferase (Gramella forsetii KT0803)
WP_026915266.1	GH18	chitinase I, partial (Pantoea dispersa)
WP_026915320.1	CE11	UDP-3-O-(3-hydroxymyristoyl) N-acetylglucosamine deacetylase (Desulfomonile tiedjei DSM 6799)
WP_026915349.1	GT66	peptidyl-prolyl cis-trans isomerase (rotamase) - cyclophilin family (Candidatus Nitrososphaera evergladensis SR1)

Continued on next page

Table S6: (continued)

Accession No.	family	Annotation
WP_026915351.1	GH18	putative peroxiredoxin (Peptoclostridium difficile)
WP_026915358.1	GT4	ABC transporter related (Desulfovibrio desulfuricans subsp. desulfuricans str. ATCC 27774)
WP_026915394.1	GH3	O-glycosyl hydrolase family protein (homolog to N-acetylglucosaminidase) (Halobacillus halophilus DSM 2266)
WP_026915411.1	CBM13	elongation factor Tu (Streptomyces albulus)
WP_026915438.1	GT2	two-component system sensor histidine kinase/response regulator, hybrid (one component system) (Bacteroides thetaiotaomicron VPI-5482)
WP_026915458.1	GH2	hypothetical protein AN2824.2 (Aspergillus nidulans FGSC A4)
WP_026915466.1	GT2	WbbL-like lipopolysaccharide biosynthesis glycosyl transferase (Gramella forsetii KT0803)
WP_026915496.1	GT4	glycosyl transferase, group 1 (Gramella forsetii KT0803)
WP_026915497.1	GT4	glycosyl transferase, group 1 (Gramella forsetii KT0803)
WP_026915498.1	GT2	glycosyl transferase, family 2 (Gramella forsetii KT0803)
WP_026915500.1	GT2	glycosyl transferase (Aequorivita sublithincola DSM 14238)
WP_026915501.1	GT9	glycosyl transferase family 9 (Lacinutrix sp. 5H-3-7-4)
WP_026915515.1	GH13-11	glycogen debranching protein (Salmonella enterica subsp. enterica serovar Enteritidis str. EC20121179)
WP_026915546.1	CBM50	LysM domain-containing protein (Zunongwangia profunda SM-A87)
WP_026915567.1	CE*	conserved hypothetical protein, secreted (Gramella forsetii KT0803)
WP_026915604.1	GH43	aldose 1-epimerase (Prevotella denticola F0289)
WP_026915611.1	GH13-7	alpha-amylase (Gramella forsetii KT0803)
WP_026915615.1	GH32	Sucrose-6-phosphate hydrolase (Klebsiella pneumoniae JM45)
WP_026915617.1	GH65	beta-phosphoglucomutase (Anabaena sp. 90)
WP_026915618.1	GH65	trehalose/maltose phosphorylase (GH65) (Formosa agariphila KMM 3901)
WP_026915619.1	GH13	alpha amylase (Gramella forsetii KT0803)
WP_026915620.1	GH13	alpha amylase (Gramella forsetii KT0803)
WP_026915663.1	CBM13	hypothetical protein; putative Ricin B lectin and Actin-like ATPase domains (Frankia alni ACN14a)
WP_026915682.1	GH96, CBM6	agarase (Thalassotalea agarivorans)
WP_026915683.1	CBM5, GH5-2	CelA8 (Pseudoalteromonas haloplanktis)
WP_026915684.1	CBM13	flavastacin (Corallococcus coralloides DSM 2259)
WP_026915685.1	CBM13	flavastacin (Corallococcus coralloides DSM 2259)
WP_026915698.1	GT2	NAD-dependent epimerase/dehydratase (Agrobacterium sp. H13-3)
WP_026915702.1	GT2	glycosyl transferase (Belliella baltica DSM 15883)
WP_026915703.1	GT2	DegT/DnrJ/EryC1/StrS aminotransferase (Pectobacterium carotovorum subsp. carotovorum PC1)
WP_026915712.1	GT2	acetyltransferase (isoleucine patch superfamily) (Rivularia sp. PCC 7116)
WP_026915716.1	GT82	bifunctional beta-1,4-N-acetylgalactosaminyltransferase/CMP-Neu5Ac synthase (Campylobacter jejuni subsp. jejuni 00-2426)
WP_026915723.1	GT2	Glycosyltransferase (Idiomarina loihiensis L2TR)
WP_026915724.1	GT4	glycosyl transferase, group 1 (Gramella forsetii KT0803)

Continued on next page

Table S6: (continued)

Accession No.	family	Annotation
WP_026915728.1	CE4	polysaccharide deacetylase (Bacteroides helcogenes P 36-108)
WP_026915729.1	GT4	glycosyl transferase, group 1 (Gramella forsetii KT0803)
WP_026915731.1	GT4	capsular polysaccharide biosynthesis glycosyl transferase (Gramella forsetii KT0803)
WP_026915732.1	GT4	glycosyl transferase possibly involved in lipopolysaccharide synthesis (Rivularia sp. PCC 7116)
WP_026915755.1	GT5	phytochrome b, partial (Mnesithea selloana)
WP_026915760.1	AA6	TrpR binding protein WrbA (Exiguobacterium sp. MH3)
WP_026915764.1	GT4	ABC transporter related (Desulfovibrio desulfuricans subsp. desulfuricans str. ATCC 27774)
WP_026915801.1	GT2, GH17	glycosyl transferase family 2 (Starkeya novella DSM 506)
WP_026915802.1	GT4	probable galactosyltransferase (Zunongwangia profunda SM-A87)
WP_026915848.1	GT2	glycosyl transferase, family 2 (Zunongwangia profunda SM-A87)
WP_026915850.1	GH5-42	glycosidase (Zunongwangia profunda SM-A87)
WP_026915851.1	GT4	glycosyltransferase (Zunongwangia profunda SM-A87)
WP_026915857.1	GT4	Mannose-1-phosphate guanylyltransferase (GDP) (Burkholderia phymatum STM815)
WP_026915867.1	GT2	transmembrane family-2 glycosyl transferase-possibly involved in biofilm formation (Gramella forsetii KT0803)
WP_026915871.1	GT2	undecaprenyl-phosphate 4-deoxy-4-formamido-L-arabinose transferase-like protein (Gramella forsetii KT0803)
WP_026915876.1	CBM14	CG33173 (Drosophila melanogaster)
WP_026915971.1	GT66	dolichyl-diphosphooligosaccharide–protein glycotransferase (Candidatus Nitrososphaera gargensis Ga9.2)
WP_026915989.1	AA*	glucoside 3-dehydrogenase (Gramella forsetii KT0803)
WP_026916000.1	GH0	hypothetical protein Celal_0179 (Cellulophaga algicola DSM 14237)
WP_026916004.1	GT20	trehalose 6-phosphate synthase/phosphatase (Gramella forsetii KT0803)
WP_026916005.1	GH15	glycosyl hydrolase, family 15 (Gramella forsetii KT0803)
WP_026916021.1	GT2	undecaprenyl-phosphate 4-deoxy-4-formamido-L-arabinose transferase (Gramella forsetii KT0803)
WP_026916029.1	GH23	membrane-bound lytic murein transglycosylase (Gramella forsetii KT0803)
WP_026916053.1	CE11	DDX20_HUMAN Probable ATP-dependent RNA helicase DDX20 (ISS) (Ostreococcus tauri)
WP_026916056.1	GT4	nicotinate-nucleotide pyrophosphorylase (carboxylating) (Flexibacter litoralis DSM 6794)
WP_026916092.1	GT4	ABC transporter related (Desulfovibrio desulfuricans subsp. desulfuricans str. ATCC 27774)
WP_026916093.1	CBM0	hypothetical protein GFO_1460 (Gramella forsetii KT0803)
WP_026916105.1	GH39	beta-xylosidase XynB (Clostridium saccharoperbutylacetonicum N1-4(HMT))
WP_026916109.1	GH72	Rab1 family GTPase (PiYpt1) putative (Albugo laibachii Nc14)
WP_026916149.1	GT2	UDP-glucose 4-epimerase (Cyanothece sp. PCC 7822)
WP_026916161.1	GT51	bifunctional transglycosylase/transpeptidase penicillin-binding protein (Zunongwangia profunda SM-A87)
WP_026916174.1	GT4	glycosyl transferase, group 1 (Gramella forsetii KT0803)

Continued on next page

Table S6: (continued)

Accession No.	family	Annotation
WP_026916224.1	GH18, GH31	hypothetical protein AN8217.2 (Aspergillus nidulans FGSC A4)
WP_026916228.1	GT49	predicted protein (Phaeodactylum tricornutum CCAP 1055/1)
WP_026916233.1	GT4	glycosyl transferase, group 1 (Gramella forsetii KT0803)
WP_026916234.1	GH3	family 3 glycosyl hydrolase/beta-lactamase fusion protein (Gramella forsetii KT0803)
WP_026916239.1	GH25	hypothetical protein SALIVB_1060 (Streptococcus salivarius CCHSS3)
WP_026916251.1	GT2	hypothetical protein SCE1572_45215 (Sorangium cellulosum So0157-2)
WP_026916254.1	GT2	transmembrane family-2 glycosyl transferase (Gramella forsetii KT0803)
WP_026916257.1	CE4	membrane protein containing polysaccharide deacetylase domain (Gramella forsetii KT0803)
WP_026916272.1	GH1	mannose-6-phosphate isomerase, class I/beta-galactosidase (Roseburia intestinalis XB6B4)
WP_026916273.1	GT83	ArnT-like undecaprenyl-phosphate alpha-4-amino-4-deoxy-L-arabinose arabinosyl transferase (Gramella forsetii KT0803)
WP_026916307.1	CE11	LpxC/FabZ bifunctional enzyme (Gramella forsetii KT0803)
WP_026916321.1	GT4	glycosyl transferase, group 1 (Zunongwangia profunda SM-A87)
WP_026916344.1	CE14	hypothetical protein GFO_1816 (Gramella forsetii KT0803)
WP_026916357.1	GH16	glycosyl hydrolase, family 16 (Gramella forsetii KT0803)
WP_026916364.1	CE3	hypothetical protein Riv_71166589 (Rivularia sp. PCC 7116)
