# Supplementary material for: Comparing polysaccharide decomposition between the type strains Gramella echinicola KMM 6050T (DSM 19838T) and Gramella portivictoriae UST040801-001T (DSM 23547T), and emended description of Gramella echinicola Nedashkovskaya et al. 2005 emend. Shahina et al. 2014 and Gramella portivictoriae Lau et al. 2005
Source: Stand Genomic Sci. 2016 Jun 3;11:37. doi: 10.1186/s40793-016-0163-9 (PMC4891872; doi:10.1186/s40793-016-0163-9)
Supplement: Additional file 2: Figure S1. — The laminarin-like PUL. Figure S2. The 1,4-linked glucose-polymer-like PUL. Figure S3. The alginate-like PUL. Figure S4. Part of the alginate-like polysaccharide decom- position pathway. Figure S5. The sulfated β-d-glucoside PUL. Figure S6. The two combined β-d-fructans PUL. Figure S7. Part of the pectin-like polysaccharide decomposition pathway. (PDF 642 kb) [file 40793_2016_163_MOESM2_ESM.pdf]

Comparing polysaccharide decomposition among the type strains *Gramella echinicola* KMM 6050^{T} (DSM 19838^{T}) and *Gramella portivictoriae* UST040801-001^{T} (DSM 23547^{T}), and emended description of *Gramella echinicola* Nedashkovskaya et al. 2005 emend. Shahina et al. 2014 and *Gramella portivictoriae* Lau et al. 2005

Irina Panschin[1], Sixing Huang[1], Jan P. Meier-Kolthoff[1], Brian J. Tindall[1], Manfred Rohde[2], Alla Lapidus[3], James Han[4], Stephan Trong[4], Matthew Haynes[4], T.B.K. Reddy[4], Marcel Huntemann[4], Amrita Pati[4], Natalia N. Ivanova[4], Konstantinos Mavromatisv[5], Victor Markowitz[5], Tanja Woyke[4], Markus Göker[1], Hans-Peter Klenk[6], Nikos C. Kyrpides[5,7] and Richard L. Hahnke[1,*]

[1] Leibniz Institute DSMZ – German Collection of Microorganisms and Cell Cultures, Braunschweig, Germany. [2] Helmholtz Centre for Infection Research, Braunschweig, Germany. [3] Algorithmic Biology Lab, St. Petersburg Academic University, St.Petersburg, Russia. [4] Genome Biology Program, Department of Energy Joint Genome Institute, Walnut Creek, California, USA. [5] Biological Data Management and Technology Center, Lawrence Berkeley National Laboratory, Berkeley, California, USA. [6] School of Biology, Newcastle University, Newcastle upon Tyne, UK. [7] School of Biology, King Abdulaziz University, Jeddah, Saudi Arabia.
*Corresponding author: Richard L. Hahnke, richard.hahnke@dsmz.de

Additional file 2 – Supporting Information – Figures

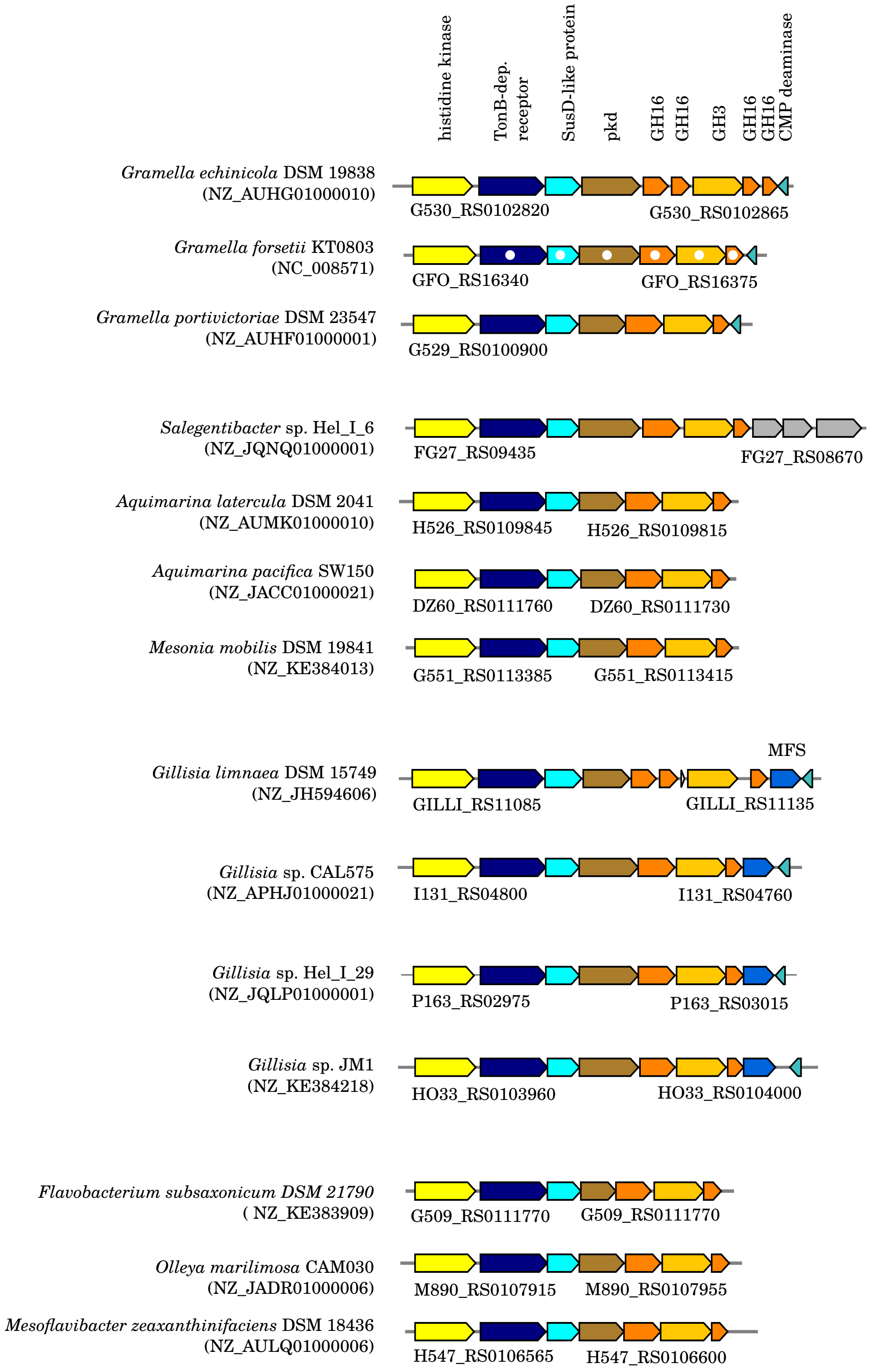

Figure S1: A laminarin-like PUL greatly conserved among *Gramella* and other closely related *Flavobacteriaceae* genera. Open circles indicate genes which were specifically expressed by '*Gramella forsetii*' KT0803 when grown on laminarin, as shown by Kabisch et al. [3]. Locus tags are given below both the first and last gene of the loci. Accession numbers in brackets are GenBank accession numbers of the corresponding contig. Investigation of syntenic loci was done using MultiGeneBlast [5]. A description of glycoside hydrolase (GH), polysaccharide lyase (PL) and carbohydrate esterase (CE) families can be seen at the CAZy homepage [1, 4]. SusD, SusD-like protein; PKD, pkd domain containing protein.

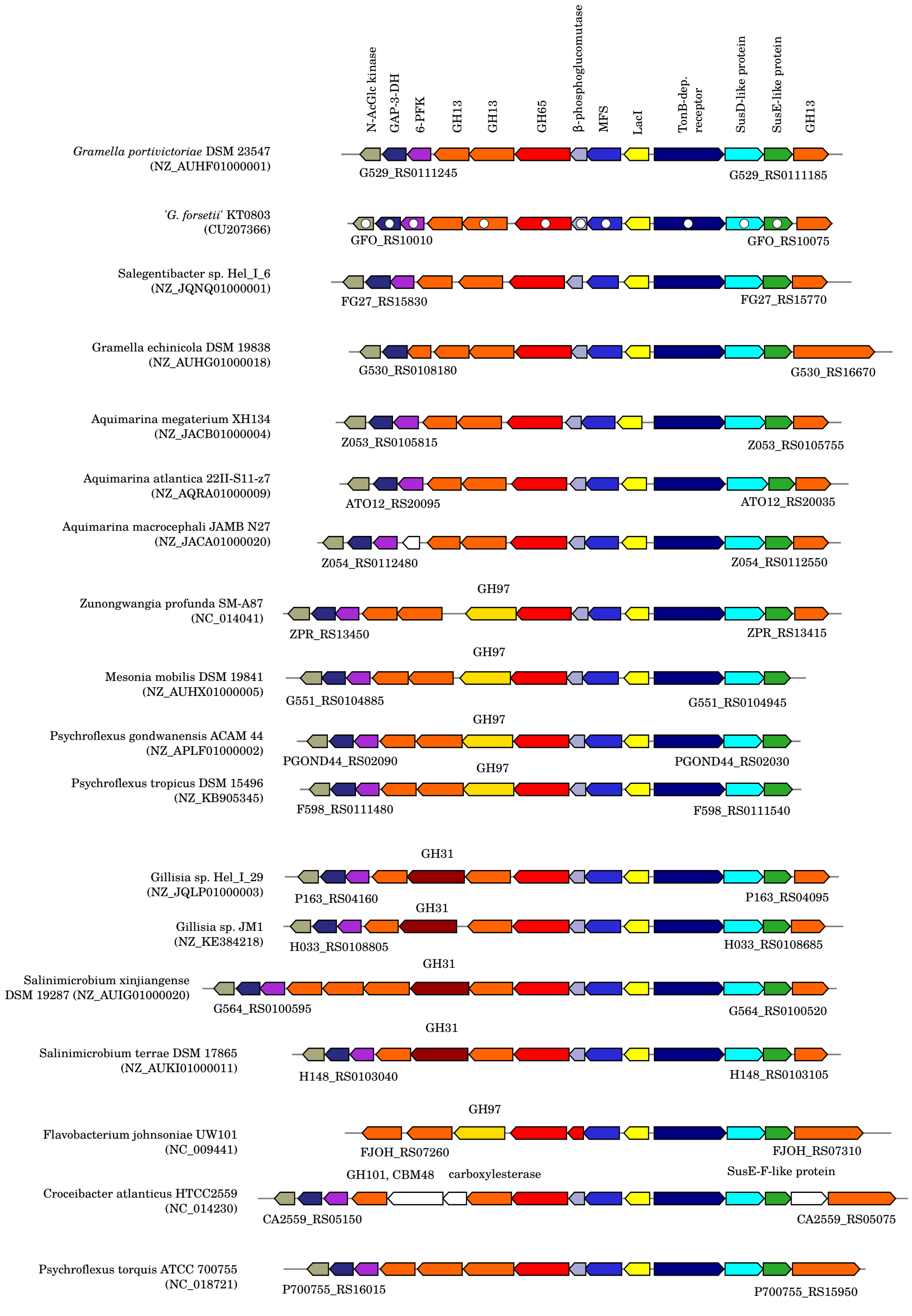

Figure S2: An 1,4-linked glucose-polymer-like PUL greatly conserved among *Gramella* and other closely related *Flavobacteriaceae* genera. Open circles indicate genes which were specifically expressed by '*Gramella forsetii*' KT0803 when grown on laminarin, as shown by Kabisch et al. [3]. Locus tags are given below both the first and last gene of the loci. Accession numbers in brackets are GenBank accession numbers of the corresponding contig. Investigation of syntenic loci was done using MultiGeneBlast [5]. A description of glycoside hydrolase (GH), polysaccharide lyase (PL) and carbohydrate esterase (CE) families can be seen at the CAZy homepage [1, 4]. LacI, LacI family transcriptional regulator; MFS, major facilitator superfamily transporter; 6-PFK, 6-phosphofructokinase; GAP-3-DH, glyceraldehyde 3-phosphate dehydrogenase; N-AcGlc, N-acetylglucosamine.

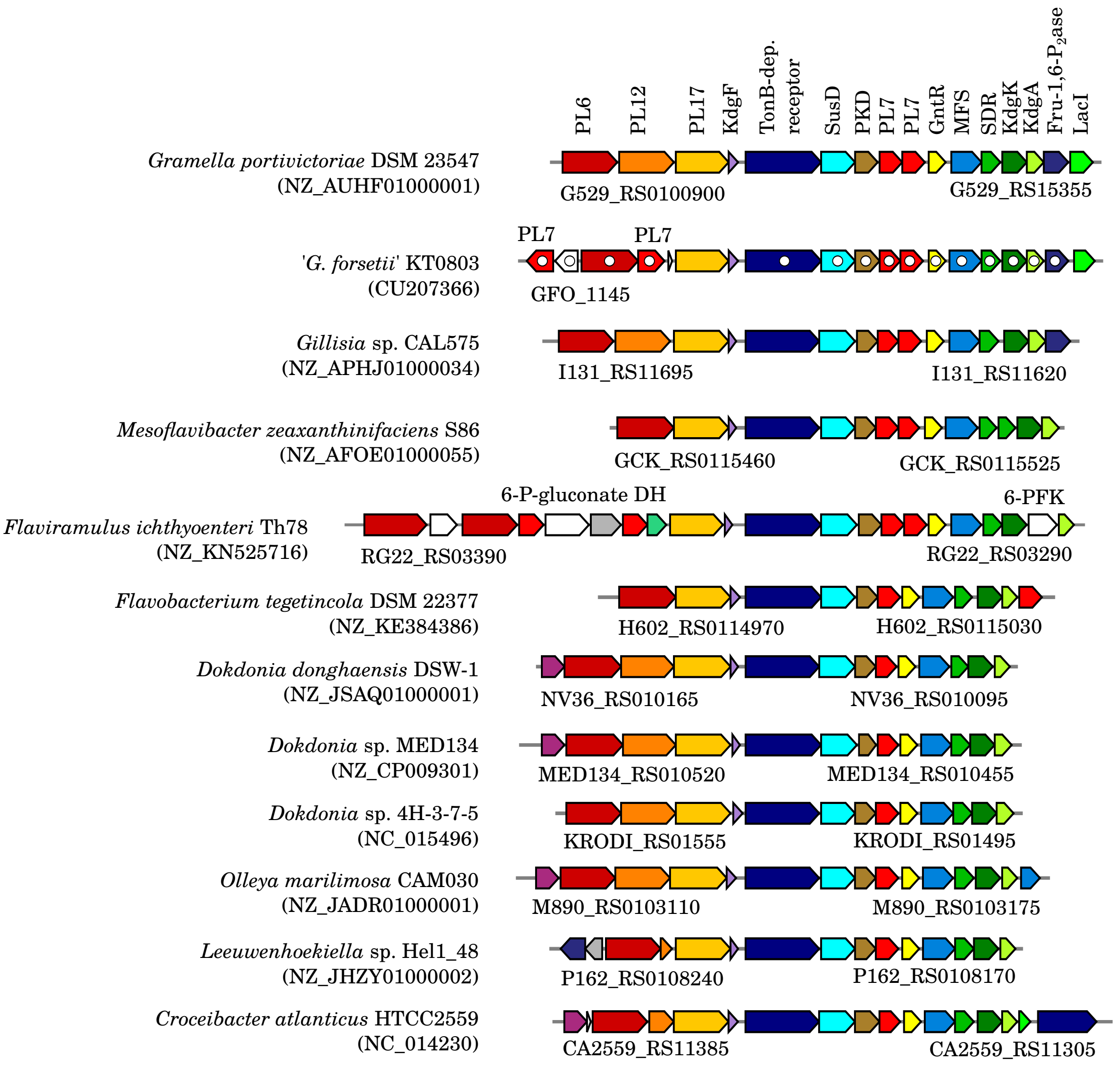

Figure S3: An alginate-like PUL of *G. portivictoriae* DSM 23547^{T}, '*Gramella forsetii*' KT0803 and other *Flavobacteriaceae*. Open circles indicate genes which were specifically expressed by '*Gramella forsetii*' KT0803 when grown on laminarin, as shown by Kabisch et al. [3]. Locus tags are given below both the first and last gene of the loci. Accession numbers in brackets are GenBank accession numbers of the corresponding contig. Investigation of syntenic loci was done using MultiGeneBlast [5]. A description of glycoside hydrolase (GH), polysaccharide lyase (PL) and carbohydrate esterase (CE) families can be seen at the CAZy homepage [1, 4]. PKD, pkd domain containing protein; SusD, SusD-like protein; GntR, GntR family transcriptional regulator; LacI, LacI family transcriptional regulator; MFS, major facilitator superfamily transporter; SDR, short-chain dehydrogenases/reductases; KdgF, pectin degradation protein; KdgK, 2-dehydro-3-deoxygluconokinase; KdgA, keto-deoxy-phosphogluconate aldolase; Fru-1,6-P2ase.

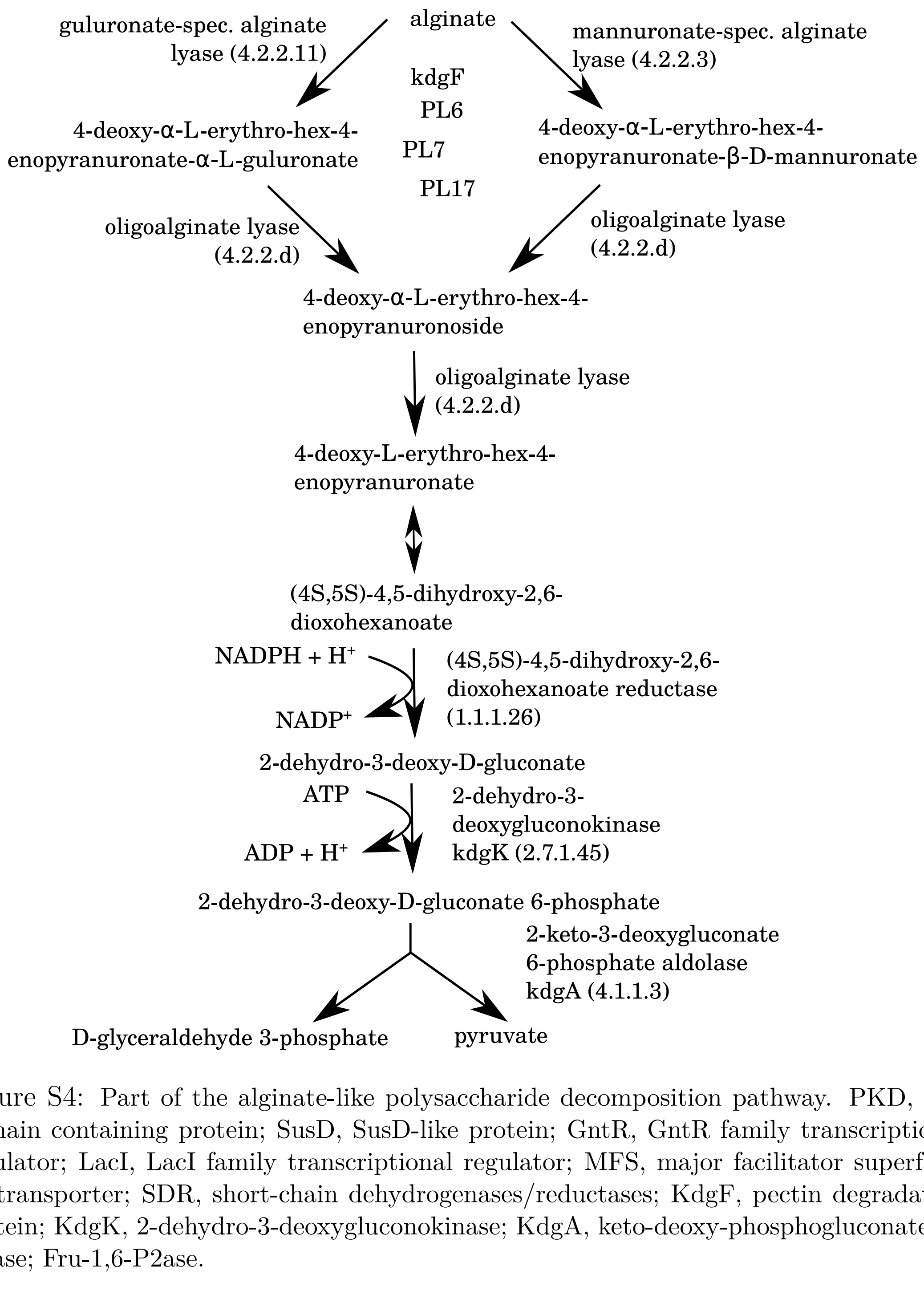

Figure S4: Part of the alginate-like polysaccharide decomposition pathway. PKD, pkd domain containing protein; SusD, SusD-like protein; GntR, GntR family transcriptional regulator; LacI, LacI family transcriptional regulator; MFS, major facilitator superfamily transporter; SDR, short-chain dehydrogenases/reductases; KdgF, pectin degradation protein; KdgK, 2-dehydro-3-deoxygluconokinase; KdgA, keto-deoxy-phosphogluconate aldolase; Fru-1,6-P2ase.

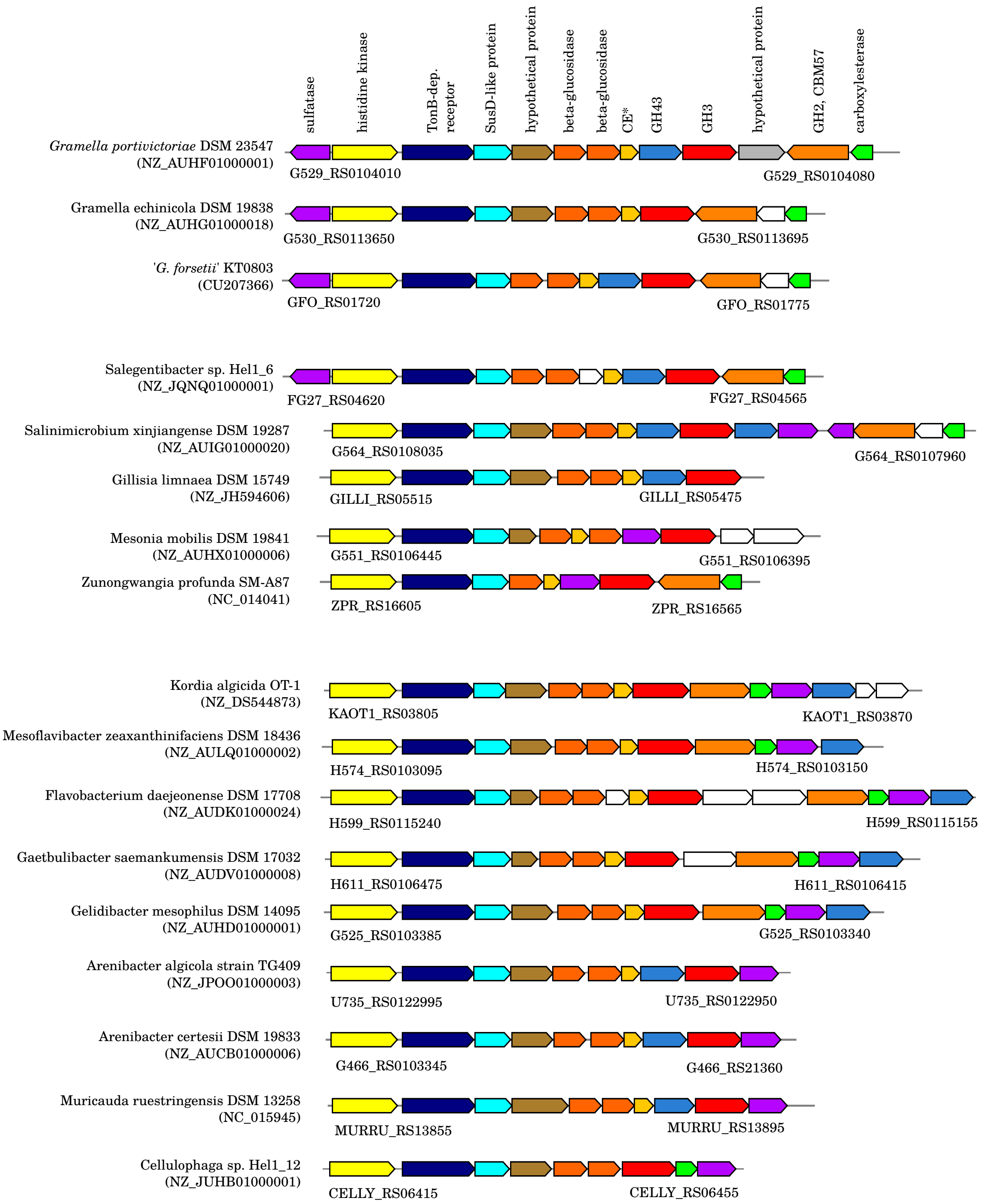

Figure S5: A sulfated β-D-glucoside PUL greatly conserved among *Gramella* and other closely related *Flavobacteriaceae* genera. Locus tags are given below both the first and last gene of the loci. Accession numbers in brackets are GenBank accession numbers of the corresponding contig. Investigation of syntenic loci was done using MultiGeneBlast [5]. A description of glycoside hydrolase (GH) and polysaccharide lyase (PL) families can be seen at the CAZy homepage [1, 4].

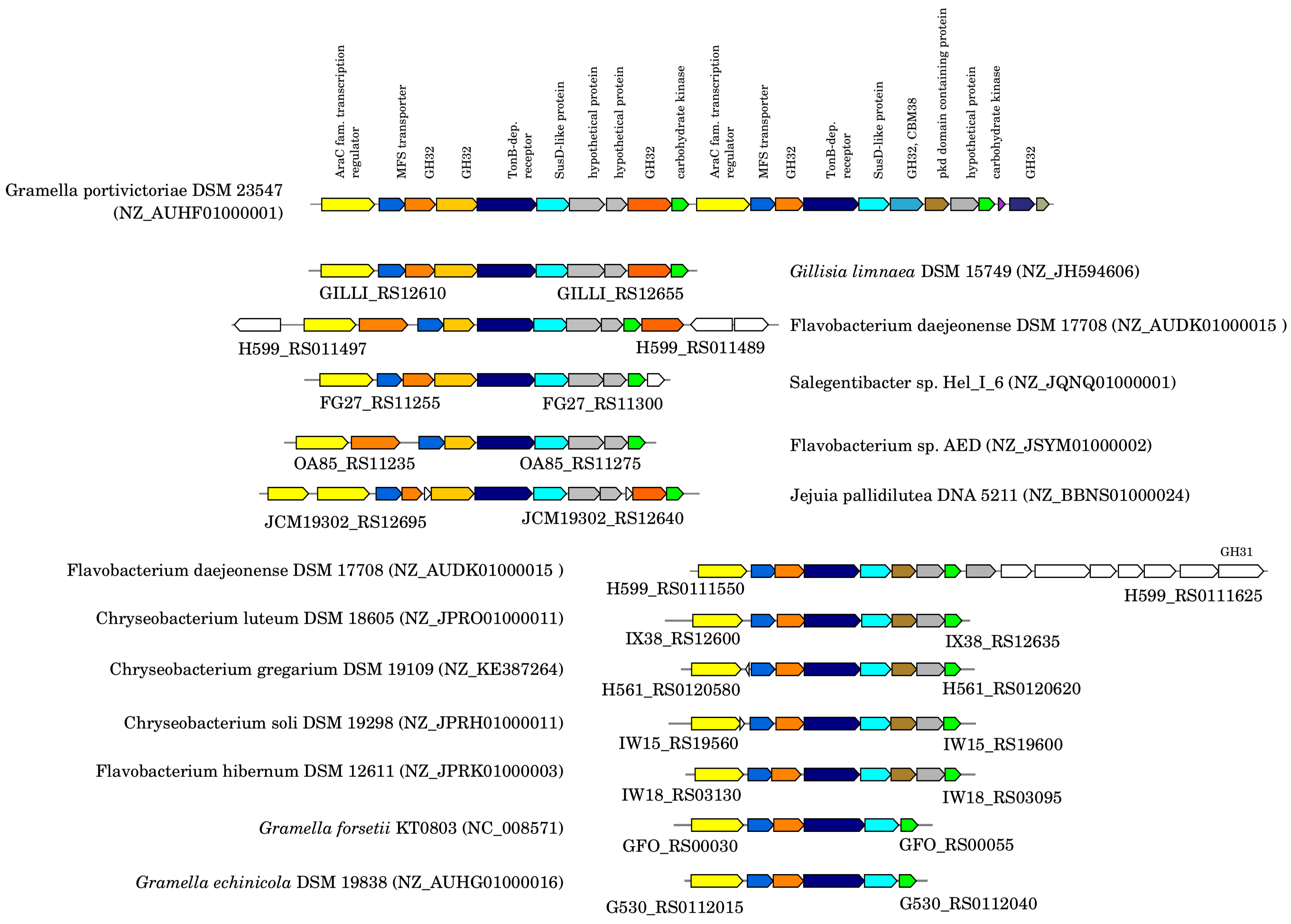

Figure S6: Two combined β-D-fructans PUL unique for *G. portivictoriae* DSM 23547^{T}. Locus tags are given below both the first and last gene of the loci. Accession numbers in brackets are GenBank accession numbers of the corresponding contig. Investigation of syntenic loci was done using MultiGeneBlast [5]. A description of glycoside hydrolase (GH) and polysaccharide lyase (PL) families can be seen at the CAZy homepage [1, 4].

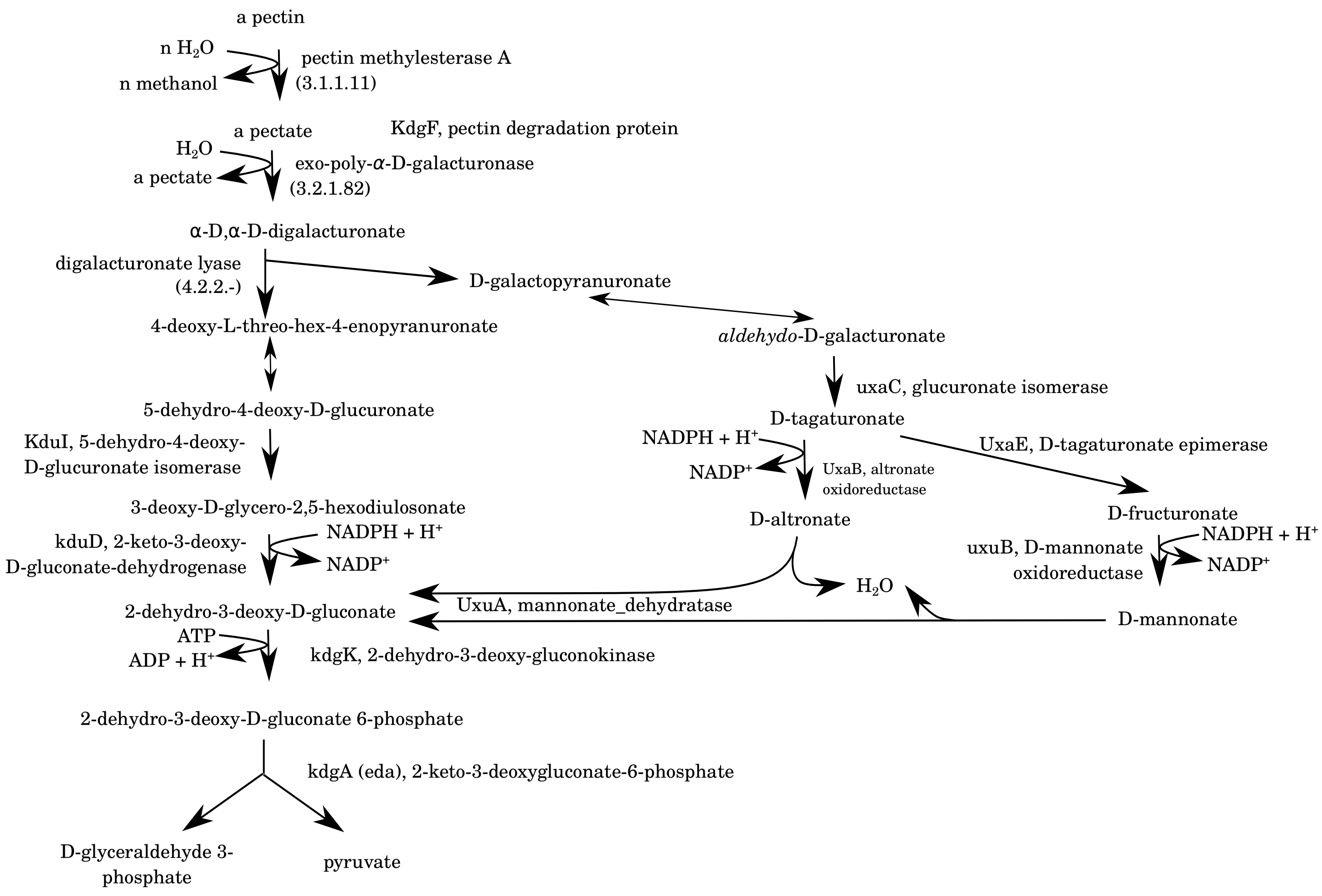

Figure S7: Part of the pectin-like polysaccharide decomposition pathway, encoded by genes of the *G. portivictoriae* DSM 23547^T genome. The full range of pathways for pectin decomposition and catabolism can be seen at MetaCyc, pathway PWY-6516 [2].

References

[1] Cantarel BL, Coutinho PM, Rancurel C, Bernard T, Lombard V, and Henrissat B. The Carbohydrate-Active EnZymes database (CAZy): an expert resource for glycogenomics. Nucleic Acids Res. 2009; 37:D233–D238.

[2] Caspi R, Altman T, Billington R, Dreher K, Foerster H, Fulcher CA, et al. The MetaCyc database of metabolic pathways and enzymes and the BioCyc collection of Pathway/Genome Databases. Nucleic Acids Res. 2014; 42:D459–D471.

[3] Kabisch A, Otto A, König S, Becher D, Albrecht D, Schüler M, et al. Functional characterization of polysaccharide utilization loci in the marine Bacteroidetes '*Gramella forsetii*' KT0803. ISME J. 2014; 8:1492–502.

[4] Lombard V, Golaconda Ramulu H, Drula E, Coutinho PM, and Henrissat B. The carbohydrate-active enzymes database (CAZy) in 2013. Nucleic Acids Res. 2014; 42:D490–D495.

[5] Medema MH, Takano E, and Breitling R. Detecting sequence homology at the gene cluster level with MultiGeneBlast. Mol. Biol. Evol. 2013; 30:1218–23.
